# An analysis of the trends in the usage of Pharmaceutical Benefits Scheme-subsidised cancer drugs in Australia from 2012 to 2022

**DOI:** 10.1007/s00432-024-05889-x

**Published:** 2024-07-31

**Authors:** Jasmine Lee, Anastasios Panagiotelis, Rose Cairns, Nial J. Wheate

**Affiliations:** 1 School of Pharmacy, Faculty of Medicine and Health, The University of Sydney, Sydney, NSW Australia; 2 The University of Sydney Business School, Sydney, NSW Australia; 3 New South Wales Poisons Information Centre, The Children’s Hospital at Westmead, Sydney, NSW Australia; 4 School of Natural Sciences, Faculty of Science and Engineering, Macquarie University, Sydney, NSW Australia

**Keywords:** Cancer, Chemotherapy, Drugs, eviQ, Medicare, Australia

## Abstract

**Purpose:**

Cancer treatment remains a significant and escalating healthcare expense worldwide. Although annual reports on total cancer care costs are available, the potential impact of evolving treatment guidelines and the introduction of new drugs on future budgeting remains largely uncertain. The aim of this study was to examine the trends in the use of Pharmaceutical Benefits Scheme (PBS)-subsidised cancer drugs in Australia over the past decade.

**Methods:**

PBS codes for all PBS-subsidised cancer drugs that were listed in government-endorsed treatment protocols were obtained and used to retrieve usage data. Their patterns of use, represented by the number of prescriptions (services) processed by Services Australia, were analysed for the period between 2012 and 2022.

**Results:**

The overall prescribing of cancer drugs is outpacing Australia’s population growth, primarily due to an ageing population and the accelerated rise in cancer diagnoses observed over the past decade. From 846 eviQ protocols, 141 cancer drugs were available on the PBS, of which kinase inhibitor (39 drugs) and monoclonal antibody drugs (24 drugs) had the highest increase in use during the study period; 16% and 23% respectively. Of the 8 drug classes, hormonal agents (20 drugs) were the most prescribed.

**Conclusion:**

The utilisation of PBS-listed cancer drugs is increasing faster than population growth, especially for high-cost monoclonal antibody and kinase inhibitor drugs, indicating continued pressure on government spending.

**Supplementary Information:**

The online version contains supplementary material available at 10.1007/s00432-024-05889-x.

## Introduction

Cancer continues to be a debilitating disease, ranking among the leading causes of death worldwide and imposing substantial health and economic challenges, particularly in high-income countries (Springer et al. 2024; Sullivan et al. 2011). This burden is reflected in Australia, where more than 100,000 individuals are diagnosed each year (Australian Institute of Health and Welfare 2023). Treatment for cancer usually takes a tailored approach and can involve any, or a combination, of surgery, radiation therapy, or drug therapies. The use of cancer drugs remains widespread, since they are often used in neo-adjuvant and adjuvant therapy, before and after surgery, and are the preferred method for the treatment of metastatic cancers (Cancer Australia 2024; Australian Institute of Health and Welfare 2023). The different types of drug therapy can include chemotherapy (cytotoxics), immunotherapy, hormonal therapy, and targeted therapy.

Established in 2009 under the auspices of the government-funded Cancer Institute New South Wales, eviQ stands as an Australian online resource aimed at bolstering nationwide cancer control amidst evolving care systems (eviQ 2024; Langton and Pearson 2011). Developed by multidisciplinary teams and drawn from evidence-based practices, its tailored protocols for diverse cancer types and stages seamlessly integrate into clinical practice to guide healthcare professionals towards optimal cancer care. While integral to the New South Wales Cancer Plan and primarily utilised within the state, eviQ’s influence extends to support physicians across the country, and now increasingly globally, with its comprehensive guidelines (Langton and Pearson 2011; Shingleton et al. 2024).

There are annual reports on the total cost of cancer care to the Australian healthcare system. In 2016, the Australian Institute of Health and Welfare (AIHW) reported that healthcare costs directly associated with cancer were an estimated 10.1 billion Australian dollars, with 9.7 billion covering diagnosis and treatment, but excluding screening services (Australian Institute of Health and Welfare 2021; Goldsbury et al. 2018). The cost of drug treatments in Australia is largely covered by the government through the Pharmaceutical Benefits Scheme (PBS), which ensures that medicines on its Schedule can be accessed and provided to patients at a government-subsidised price (Services Australia 2023). In fact, most public patients treated under the Australian healthcare system are provided drugs free of charge or at a maximum price of around $30 (Services Australia 2022). For 2021 and 2022, $14.7 billion was spent on PBS expenditure. Drugs for cancer accounted for 16 of the top 50 highest-costing medicines at a total cost of $2.7 billion. Monoclonal antibodies and kinase inhibitors accounted for 12 of the 16 drugs at a cost of $1.8 billion (Services Australia 2022; Hernandez et al. 2018).

While broader trends in cancer costs are available, they have yet to be analysed at the level of drug classes and specific agents. This paper aimed to examine temporal patterns in the use of PBS-subsidised cancer drugs in Australia by both drug class and by individual agent spanning 2012–2022.

## Methods

### eviQ protocols

To identify all chemotherapy (cytotoxic), hormonal, immuno, and targeted therapy drugs used to treat cancer in Australia, all protocols from the following categories on eviQ were searched between 6 and 10 October 2023: medical oncology (including breast, colorectal, gynaecological, head and neck, neurological, respiratory, sarcoma, skin, upper gastrointestinal, urogenital, and rare cancers), and haematology (including leukaemias, lymphoma, and multiple myeloma). Where a drug was intended to treat the side effects of cancer treatment, it was excluded from the collected list.

### PBS codes

The PBS Schedule was searched between 11 and 13 October 2023 to retrieve all PBS codes currently in use for each drug; the list of codes is provided in supplementary material. A drug not listed on the Schedule meant that it was not currently subsidised for use by the government and was subsequently excluded from the list for analysis; these excluded drugs are detailed in supplementary material. Where a drug had multiple PBS codes, including ones associated with alternative formulations not used in cancer treatment, they were cross-referenced with eviQ protocols and the Australian Medicines Handbook (AMH), and consequently removed if found irrelevant to cancer treatment. Examples included topical fluorouracil for skin conditions and dexamethasone for eye conditions.

### Medicare statistics

Publicly available data on the use of cancer drugs was retrieved by searching the Australian Department of Human Services’ PBS Item Reports (Services Australia 2024a; b) using the relevant PBS codes. Usage was reported by “number of services” where each service represented a prescription dispensed and processed by Services Australia between January 2012 and December 2022, inclusive. A report was generated for each PBS code, or group of codes, pertaining to the same drug, strength, and quantity.

### Data extraction and analysis

Drugs were initially grouped based on the 18 antineoplastic categories outlined in the AMH. These initial groups were then recategorised by merging those that had similar mechanisms of action. For example, platinum-based drugs including carboplatin, cisplatin, and oxaliplatin were moved from their separate category of “platinum-based compounds” and combined with “alkylating agents”. A resulting eight drug classes were subsequently used for this analysis: alkylating agents, antimetabolites, hormonal agents, kinase inhibitors, mitotic inhibitors, monoclonal antibodies, other agents, and topoisomerase inhibitors. Data were imported into Excel, where the average annual percentage change (APC) and 95% confidence interval from 2012 to 2022 were calculated for each drug class. All statistical analyses were conducted using R version 4.3.2 (R Core Team 2023). Visual plots for the drug services for all classes throughout the 10-year span were generated using the ‘tidyverse’ package version 2.0.0 (Wickham et al. 2022).

### Population and cancer rate data

Data on the population of Australia between the years 2012 and 2022, inclusive, were obtained from the Australian Bureau of Statistics (ABS), and statistics on the number of cancer patients for the same years was retrieved from the AIHW (Australian Bureau of Statistics 2023; Australian Institute of Health and Welfare 2023).

## Results

A total of 846 eviQ protocols were searched, of which there were 625 medical oncology protocols and 221 haematology protocols. There were 955 relevant PBS codes in total. Of these, 880 returned data on services while 75 did not have any subsidised use during the study period. After removing non-PBS-listed cancer drugs, and drugs that did not have any codes that gave data from the PBS Item Reports between 2012 and 2022, there were 141 final drugs included in the analysis. The full list is presented in Table 1. The average APCs and associated 95% confidence intervals for each drug class over the 10-year period from 2012 to 2022 are presented in Table 1.

**Table 1:** Cancer drugs included on the Australian PBS and their annual percentage change (APC) in use from 2012 to 2022

Drug class (141)	Drugs				APC (95% CI)
Monoclonal antibodies (24)	Atezolizumab Avelumab Bevacizumab Blinatumomab Brentuximab vedotin Cemiplimab Cetuximab	Daratumumab Denosumab Durvalumab Elotuzumab Gemtuzumab ozogamicin	Inotuzumab ozogamicin Ipilimumab Nivolumab Obinutuzumab **Ofatumumab Panitumumab	Pembrolizumab Pertuzumab Rituximab Sacituzumab govitecan Trastuzumab Trastuzumab emtansine	23.0% (16.9–29.1)
Kinase inhibitors (39)	Abemaciclib Acalabrutinib Afatinib Alectinib Axitinib Binimetinib Brigatinib Cabozantinib *Ceritinib	Cobimetinib *Crizotinib Dabrafenib Dasatinib Encorafenib Entrectinib *Erlotinib *Gefitinib Gilteritinib Ibrutinib	Idelalisib Imatinib Lapatinib Lenvatinib Lorlatinib Midostaurin Nilotinib Osimertinib Palbociclib Pazopanib	Ponatinib Ribociclib Ripretinib Ruxolitinib Sorafenib Sunitinib Tepotinib Trametinib Vemurafenib Zanubrutinib	16.4% (9.1–23.7)
Other agents (18)	Arsenic trioxide (ATO) Bleomycin Bortezomib Carfilzomib Dexamethasone	Everolimus Lenalidomide Niraparib Olaparib Pamidronate	Pomalidomide Selinexor Sonidegib Thalidomide Venetoclax	Vismodegib Vorinostat Zoledronic acid	6.5% (1.5–11.6)
Mitotic inhibitors (8)	Cabazitaxel Docetaxel	Eribulin Nab-paclitaxel	Paclitaxel Vinblastine	Vincristine Vinorelbine	11.5% (− 1 to 23.9)
Antimetabolites (14)	Azacitidine Capecitabine Cladribine Cytarabine	Fludarabine Fluorouracil Gemcitabine Mercaptopurine	Methotrexate Pemetrexed Pralatrexate	Raltitrexed Tioguanine Trifluridine with tipiracil	5.3% (1.9–8.6)
Topoisomerase inhibitors (8)	Etoposide Doxorubicin	Doxorubicin liposomal Epirubicin	Idarubicin Irinotecan	**Mitoxantrone Topotecan	4.2% (− 0.7 to 9.1)
Alkylating agents (10)	Bendamustine Carboplatin Carmustine	Chlorambucil Cisplatin Cyclophosphamide	Ifosfamide Melphalan Oxaliplatin	Temozolomide	3.7% (0.3–7.0)
Hormonal agents (20)	Abiraterone Anastrozole Apalutamide Bicalutamide Cyproterone	Darolutamide Degarelix Enzalutamide Exemestane *Flutamide	Fulvestrant Goserelin Goserelin with bicalutamide Lanreotide (Somatuline autogel) Letrozole	Leuprorelin Leuprorelin with bicalutamide Octreotide long-acting release (LAR) Tamoxifen Triptorelin	2.3% (− 4.2 to 8.8)

*APC* annual percentage change

*Denotes drugs included in superseded eviQ cancer treatment protocols

**Denotes drugs included in discontinued eviQ cancer treatment protocols

Between the end of 2011 and 2021, Australia experienced 15% population growth, with the population rising from 22.9 to 26.3 million. Concurrently, cancer rates surged from 125,813 to 160,570 diagnoses per year, marking a larger 28% increase, and the population aged 65 and above grew from 3.3 to 4.5 million: a growth of 73% (Australian Bureau of Statistics 2023; Australian Institute of Health and Welfare 2023). As a result, the population-adjusted cancer diagnosis rate surged by approximately 60.80 per 100,000 individuals during this period. This suggests a swifter rise in cancer incidence when compared with population growth, which aligns with demographic aging, as cancer is generally more prevalent among older age groups (Springer et al. 2024).

**Fig. 1:**
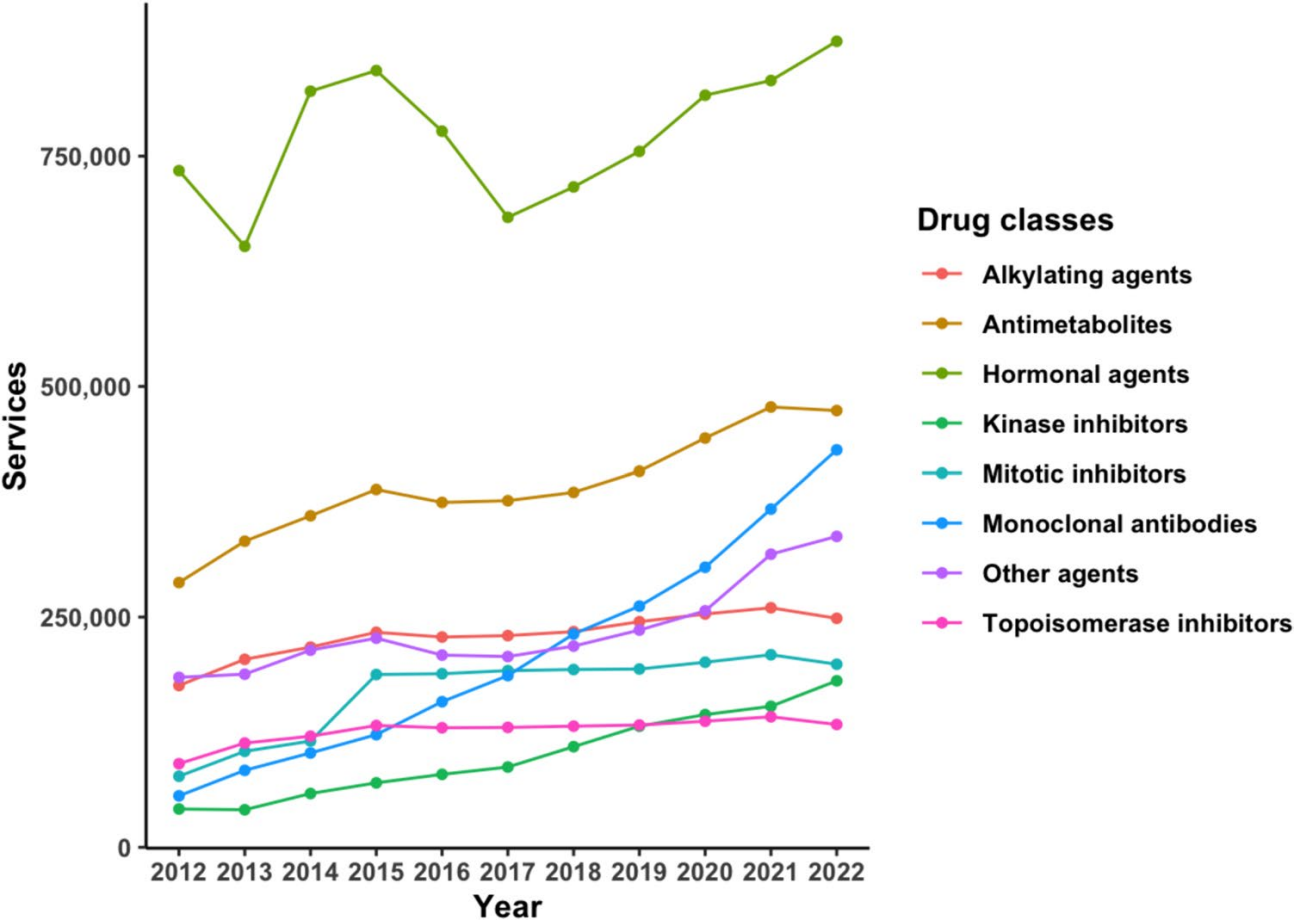
Services of PBS cancer drugs by class in Australia, 2012–2022

Our data analysis indicates a noticeable upward trend in the demand for PBS cancer drugs across Australia. An overall APC increase of 10.3% (95% CI = 5.4–15.2%) was observed for the services of all drugs, with some drugs exhibiting higher demand when compared with others at different points in time over the past 10 years. Average APCs for the 10-year period were positive for all drug classes, varying in magnitude from 2 to 23%, as shown by the trends depicted in Fig. 1.

Antimetabolites, which comprised mostly conventional chemotherapeutic drugs, had consistently high usage rates that rose from 250,000 to almost 500,000 services annually in later years (equivalent to 1000–2000 services per 100,000 head of population). This is largely due to the extensive use of fluorouracil, which was found in many protocols across different types of cancer; 1,781,098 prescriptions for fluorouracil were dispensed between 2012 and 2022, and 97 (11%) of eviQ cancer treatment protocols included the use of the drug.

Alkylating agents and topoisomerase inhibitors both showed average annual percentage changes (APCs) of approximately 4% and displayed nearly identical usage patterns, as depicted in Fig. 1. However, it is evident that the rate of increase in their usage was slower when compared with drugs in other classes and also slower when compared with the annual rate of cancer diagnoses over the same years. The use of mitotic inhibitors over the years closely resembles that of alkylating agents and topoisomerase inhibitors, except for a significant surge in 2015 following the only PBS listing this study could find for docetaxel, which occurred in 2014. Docetaxel services rose from 710 in 2014 to 31,693 in 2015 and continued to remain high alongside paclitaxel, resulting in a higher APC of 11% for mitotic inhibitors.

“Other agents” which comprised drugs outside of the named seven classes, displayed a minimal increase in use over the 10-year period, with the exception of a surge post-2020, leading to an average APC of 6.5% (Fig. 1). Throughout this period, the total services for this class generally remained below 250,000 (equivalent to 1000 per 100,000 head of population), until beyond 2020. The notable spike following 2020 can be attributed to the introduction of bortezomib, which was extensively used, onto the PBS Schedule starting from 2021. Dexamethasone was the most frequently dispensed drug within the class, representing almost 45% of the total services (1,136,192 out of 2,596,369). Within eviQ, it is primarily found in haematology treatment protocols.

### Hormonal agents

**Fig. 2:**
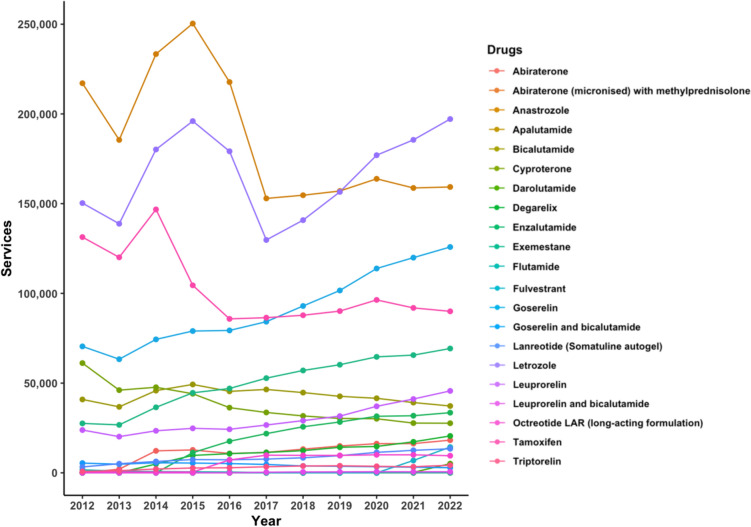
Services of hormonal cancer agents on the PBS from 2012 to 2022

Hormonal agents, though used the most, exhibited the most variability in use over the course of the study, characterised by large fluctuations between 2012 and 2017, but with the lowest overall APC of 2% (Fig. 1). The number of services in this period ranged between 625,000 and 875,000 (or 2500–3500 services per 100,000 head of population) whereas the number for every other class generally fell below 500,000 services per year (or 2000 prescriptions per 100,000 head of population). Anastrozole, letrozole, and tamoxifen emerged as the most frequently used drugs and accounted for the majority of these services (Fig. 2). Consequently, the overall changes in trend reflect the individual changes observed for these three agents.

The high usage rates for these drugs are explained by their daily use as oral formulations; the recommended dosing regimen for all three hormonal agents in the eviQ protocols is long-term, once-daily use. Anastrozole when used in breast adjuvant and metastatic chemotherapy is dosed every day for up to ten years or until disease progression. Similarly, letrozole is used in adjuvant endocrine therapy and is dosed once a day for a total of 5–10 years. In contrast, the majority of other cancer medications are administered once weekly to once every three weeks.

From 2012 to 2013, the use of hormonal agents saw a drop as the total number of services decreased from 734,254 to 652,082 (Change: − 11%). The number of anastrozole services decreased from 217,089 to 185,514, contributing to most of the overall drop, while letrozole decreased from 150,315 to 138,805, and tamoxifen from 131,368 to 120,089.

In the following year (2014), hormonal agents saw their steepest increase in use, driven by a change in the use of anastrozole (26% increase), letrozole (30%), and tamoxifen (22%). In total, services rose from 652,082 to 820,506 (or from about 2800 to 3500 per 100,000 head of population); an overall increase of 25%. This occurred after the inclusion of these medications into eviQ protocols for metastatic breast cancer in June 2012, expanding their use beyond adjuvant breast cancer therapy. This also followed the expiration of their patents, which began with anastrozole in 2010 (Qin et al. 2019).

While the use of anastrozole and tamoxifen slowed considerably from 2016, there was a notable increase in the use of goserelin and exemestane, with goserelin services surpassing tamoxifen services after 2017. Also used in the treatment of breast cancer, goserelin has been found to protect against ovarian failure during adjuvant chemotherapy (Moore et al. 2015). The eviQ protocol that combined goserelin and exemestane for breast adjuvant chemotherapy was also endorsed in late 2015.

### Monoclonal antibodies

**Fig. 3:**
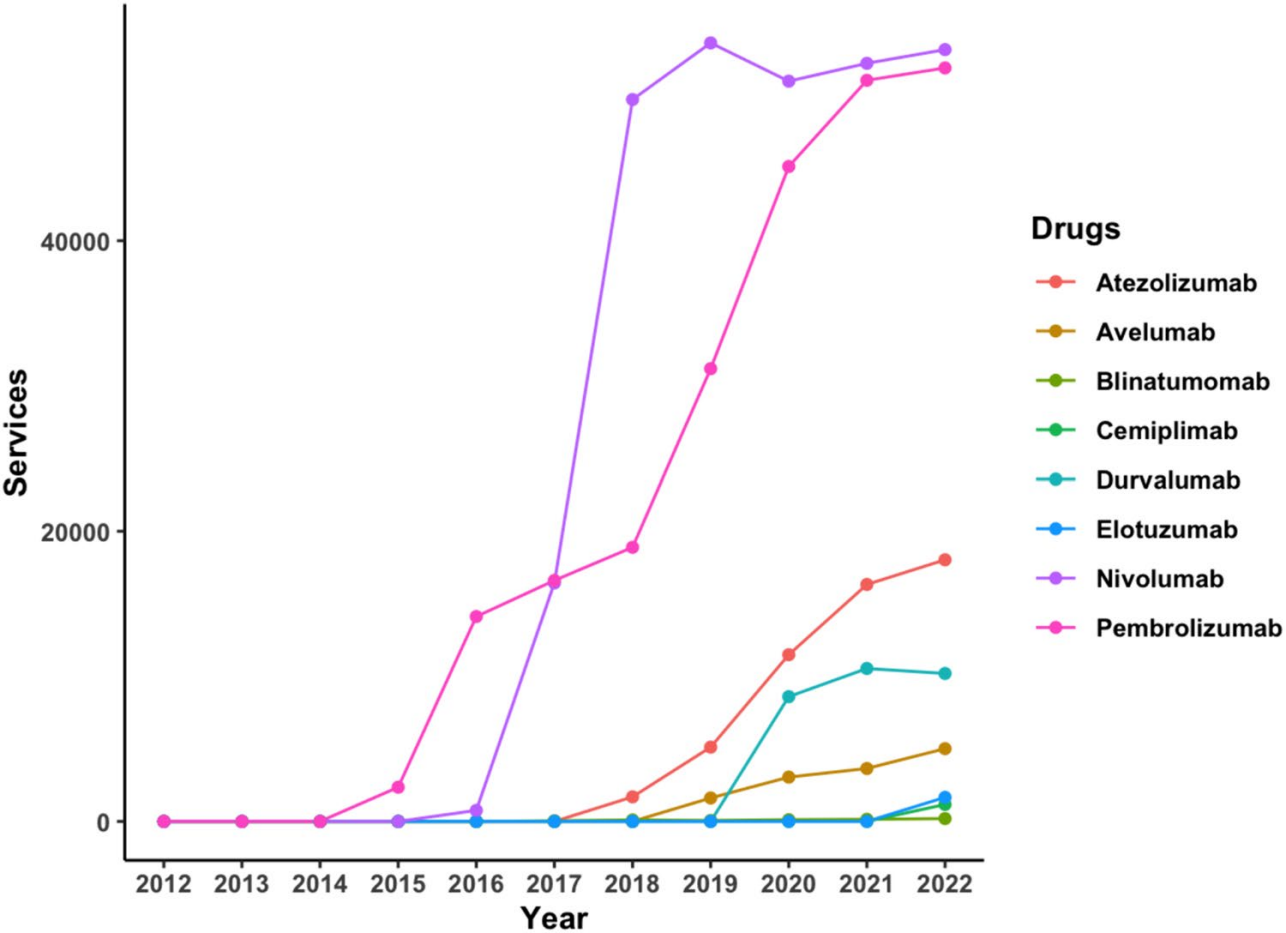
Services of immunotherapy monoclonal antibodies for cancer on the PBS from 2012 to 2022

**Fig. 4:**
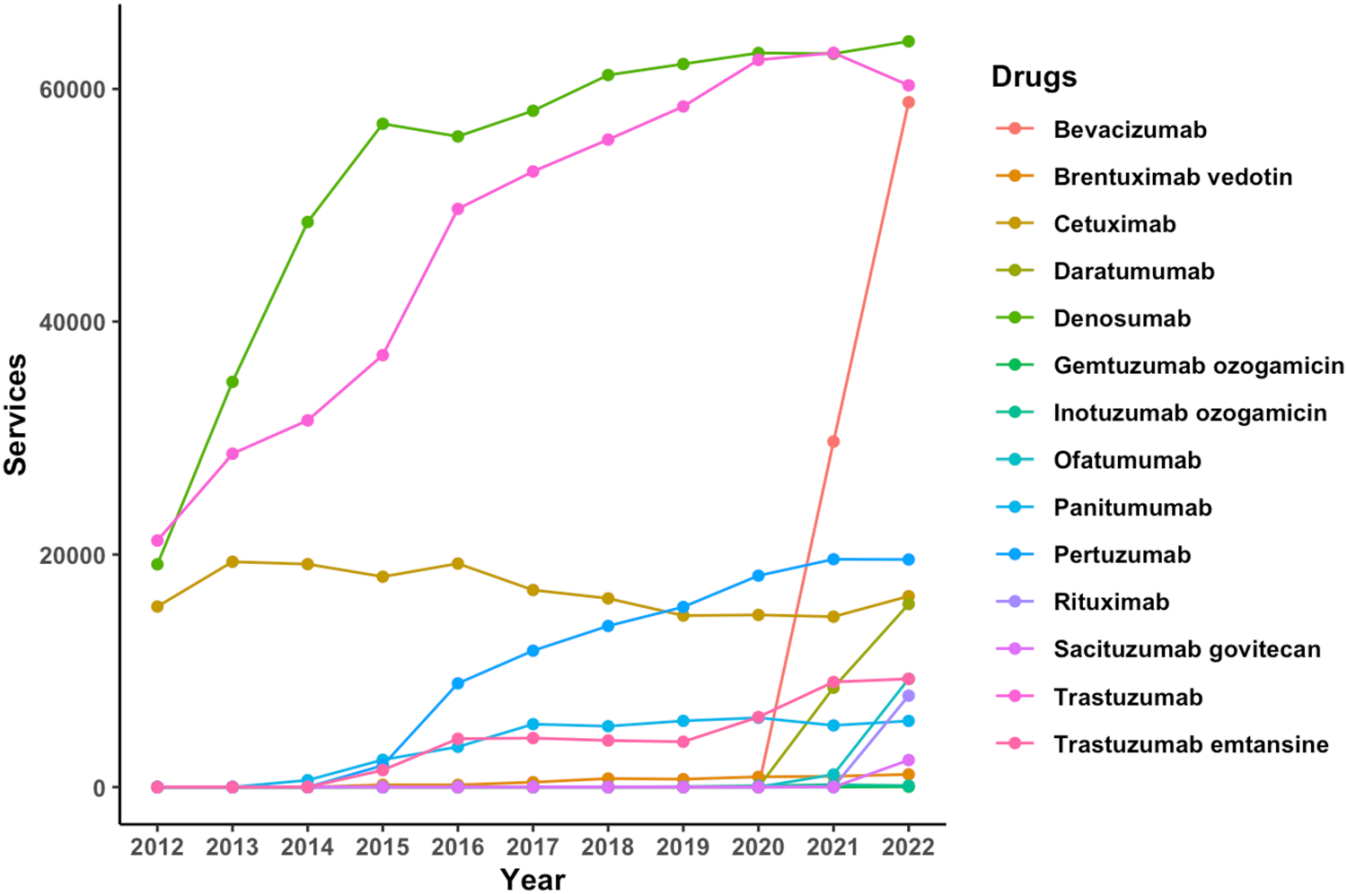
Services of targeted monoclonal antibodies for cancer on the PBS from 2012 to 2022

Of particular interest are the usage trends of the newer targeted therapy medicines, which comprised drugs grouped under “monoclonal antibodies” and “kinase inhibitors”. Of the 23 monoclonal antibodies listed within the eviQ protocols, 14 were PBS listed with data available from, or after, 2016. The year with the most newly listed agents was 2022. Monoclonal antibodies were the only drug class to consistently increase in the number of services without any decline throughout the entire period. Its average APC increase over the 10 years was the highest at 23%. Over the course of the analysis period, denosumab (APC: 15%), trastuzumab (APC: 12%), nivolumab (APC: 42%), pembrolizumab (APC: 26%), and bevacizumab (APC: 98%) stood out for having the most notable increases in use (Figs. 3 and 4). These surges were observed in the initial years following the availability of their PBS data, with bevacizumab becoming accessible only in 2021. In 2022, denosumab and trastuzumab recorded the number of services above 60,000 (or approximately 3000 per 100,000 head of population), while the remaining three drugs recorded services above 50,000 (approximately 2500 per 100,000 head of population).

Trastuzumab was one of the earliest monoclonal antibody agents to be listed on the PBS in 2012. Aside from its consistent increase in use from 2012 to 2022, with the greatest surge between 2015 and 2016 (APC: 34%), it was also one of the most used drugs across the period. Its 521,074 services amounted for 23% of the total services (*n* = 2,304,533) for all monoclonal antibodies. Trastuzumab was predominantly found in eviQ treatment protocols for breast cancer (*n* = 32 protocols; 27% of all breast cancer protocols) and used both alone and in conjunction with other drugs. Beyond its use in multiple myeloma, denosumab was predominantly used to treat a rare bone tumour, and bone metastases arising from breast and prostate cancers. Nivolumab, pembrolizumab, and bevacizumab covered a wider range of cancer treatment protocols, including those within the respiratory, neurological, and gynaecological systems, among others.

Of the 846 cancer treatment protocols retrieved from eviQ, 194 protocols included a monoclonal antibody. Among these, only 67 protocols (35%) recommended its use as standalone therapy, which meant that they were primarily used with other agents to complement existing treatment protocols. However, as many of these combinations comprised other monoclonal antibodies and kinase inhibitors, they could be replacing drugs from other classes. For example, although the FOLFIRI (modified) protocol that included the drug aflibercept was discontinued, the drugs bevacizumab, cetuximab, and panitumumab (three targeted monoclonal antibody agents) continue to be included in three other modified FOLFIRI protocols for the treatment of metastatic colorectal cancer.

### Kinase inhibitors

**Fig. 5:**
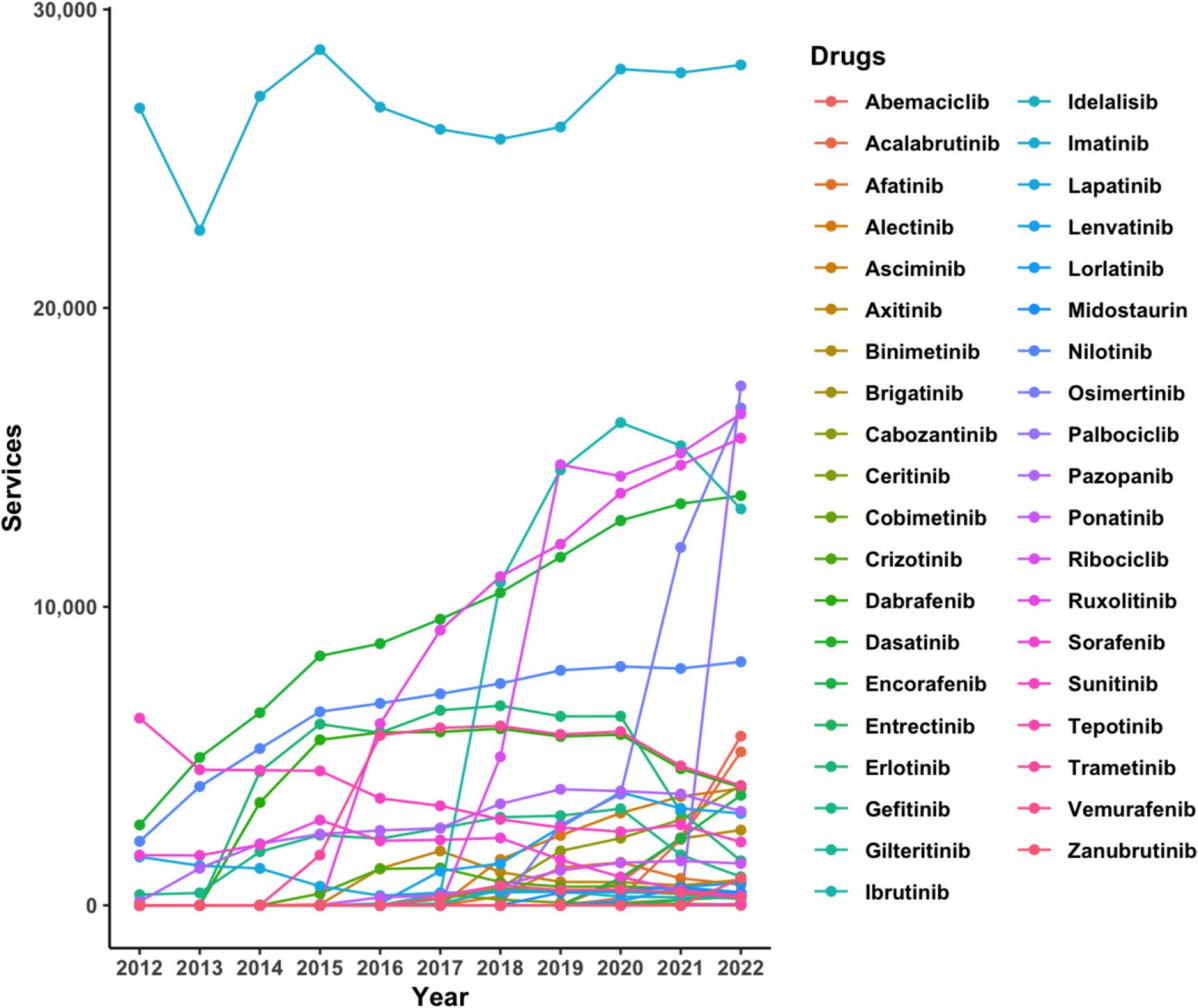
Services of kinase inhibitor cancer drugs on the PBS from 2012 to 2022

Following monoclonal antibodies, kinase inhibitors exhibited the second-highest overall APC (16%) among all the drug classes analysed. Use of drugs within this category solely increased each year from 2013 onward (Fig. 5). At the beginning of the analysis period (2012), kinase inhibitors were used the least, with only 41,596 services recorded, largely due to most agents not yet being listed on the PBS. Of the 39 kinase inhibitors listed in eviQ guidelines, 25 were PBS listed with data available from 2016, and the year 2020 saw the most newly listed agents. Subsequent years resulted in rapid increases in their use as more agents were introduced, surpassing the total services per year of topoisomerase inhibitors after 2019.

Although kinase inhibitors were not as extensively used as monoclonal antibody drugs, with services consistently below 200,000 (or 1000 per 100,000 head of population) annually, the overall usage trend mirrored that of monoclonal antibodies in this analysis. Most of the monoclonal antibodies and kinase inhibitors were listed on the PBS from the late 2010s, and this is visually reflected in the steeper rise over the 10-year period. As a result, prior to 2017, the number of services for both classes that comprised newer drugs was relatively low. From 2018, the use of drugs in both the monoclonal antibody and kinase inhibitor classes began to increase at a faster rate than was observed for the other six drug classes (Fig. 1).

Imatinib was the most frequently used kinase inhibitor in this analysis (*n* = 293,498 total services from 2012 to 2022) despite it being listed in only six eviQ protocols: for the treatment of acute lymphoblastic and chronic myeloid leukaemia, and gastrointestinal stomal cell tumours.

Out of the 846 cancer treatment protocols sourced from eviQ, 67 protocols incorporated a kinase inhibitor into the treatment regimen. In contrast to monoclonal antibodies, the majority of these protocols (58 out of 67; 87%) recommended its use as monotherapy, particularly in lung cancer treatment. Among the few instances where it was used in combination with other drugs, the additional drugs were predominantly monoclonal antibodies, such as lenvatinib and pembrolizumab for advanced endometrial and renal cell carcinomas.

## Discussion

This study found that the use of subsidised cancer drugs in Australia has increased at a pace surpassing population growth, and that the rate of increase varied substantially by class and individual agent.

Medications prescribed for long-term use in common cancers, such as hormonal drugs for breast cancer, are clearly in high demand but are also subject to significant variability, suggesting that they could potentially be challenged by emerging alternatives, or future evidence for superior alternatives. While conventional chemotherapy drugs comprising the antimetabolites, alkylating agents, topoisomerase inhibitors, and mitotic inhibitors continue to see widespread use, their prescribing appears to be only growing slowly over time. Conversely, monoclonal antibody and kinase inhibitor drugs are on the rise, and this is particularly evidenced by their rapid increase in use over the later years of this study.

Monoclonal antibodies and kinase inhibitors are widely used targeted drugs that are designed to interfere with the growth of cancer cells by targeting specific molecules or pathways (Andrew et al. 2016; Lee et al. 2018; Pérez-Herrero and Fernández-Medarde 2015; Soldi et al. 2023). Due to their specificity, they are less likely to harm non-cancerous cells and cause the side effects that are characteristic of conventional chemotherapeutic cytotoxic drugs (Pérez-Herrero and Fernández-Medarde 2015; Soldi et al. 2023). However, they are difficult and costly to manufacture, as monoclonal antibody-based agents require a rigorous process that involves recombinant DNA (rDNA) technology and mammalian cell systems to produce large quantities (Mahal et al. 2021; Posner et al. 2019). The lack of lower cost biosimilars compared with the branded antibody, as well as prescriber preference for established brands, also contributes to the growing costs of clinical demand (Grilo and Mantalaris 2019; Kaida-Yip et al. 2018; Posner et al. 2019). Following the clinical success of high-cost targeted therapy drugs in various cancers such as breast, colorectal, and lung cancers, it is likely there will be further increases in PBS cancer drug costs in coming years, due to monoclonal antibody therapy.

The development of kinase inhibitors has allowed for a paradigm shift away from relying on intravenously delivered anticancer treatments toward orally administered medicines that are also better targeted to cancers (Shen et al. 2014). Imatinib, the first approved drug in the class, effectively transformed previously fatal chronic myeloid leukaemia into a treatable condition (Cohen et al. 2021). With some 500 protein kinases present as potential drug targets in the human body, around half of which influence cancer signalling pathways, kinase inhibitors have been recognised for their importance in targeted cancer therapy. However, like monoclonal antibody-based agents, advancements in profiling techniques to produce, assess, and improve their selective efficacy remain a costly endeavour (Cohen et al. 2021; Ferguson and Gray 2018; Shen et al. 2014).

Monoclonal antibodies and kinase inhibitors emerged from this analysis as the classes with the most substantial growth in use over the 10-year period, particularly in the more recent years. In addition to those currently in use and incorporated in eviQ protocols, there are also newer agents that have been approved by the Australian Therapeutic Goods Administration (TGA) that are expected to be considered for PBS inclusion in accordance with clinical demand and cost-effectiveness analyses. Examples include relatlimab, used in combination with nivolumab to treat melanoma, and regorafenib, in the treatment of advanced colorectal cancer (eviQ 2024). Although approved by the TGA for use and currently established in eviQ protocols for their respective areas of cancer treatment, these agents are not yet listed on the PBS Schedule. If added, they would be expected to push PBS expenditure up further.

Despite accounting for a substantial portion of the annual expenditure on PBS cancer treatment drugs (67%), the volume of PBS-subsidised prescriptions (services) for monoclonal antibodies and kinase inhibitors, crucial for targeted therapy, remained notably lower than any of the top 50 most frequently prescribed PBS drugs for 2021 and 2022. This highlights their high cost relative to other drugs. In most cases, targeted cancer drugs incur costs in the thousands of dollars or above, whereas conventional chemotherapy drugs usually fall within the tens to hundreds of dollars per treatment cycle. As of May 2024, traditional, non-targeted therapy medications are relatively inexpensive to the government; for example, anastrozole is $34.95 for the dispensed price of the maximum quantity (DPMQ) of 30 tablets per month. In contrast, the dispensed price of the maximum amount (DPMA) of blinatumomab, a monoclonal antibody drug, is currently $66,317.34 for a single vial. Ibrutinib 140 mg capsules, the frequently used kinase inhibitor drug, were priced at $7953.68 in May 2024, but when taken at the recommended dose of 560 mg daily for mantle cell lymphoma, each treatment cycle is expected to cost around $9880. Considering that the APCs for monoclonal antibodies and kinase inhibitors were the highest among all drug classes in this analysis, with increases of 23% and 16% respectively, it is anticipated that their continued utilisation in cancer treatment in the coming years will consequently lead to higher associated PBS costs to governments.

As patent expirations pave the way for lower cost biosimilars and generics to enter the market, increased use of these agents in targeted cancer therapy is anticipated due to improved accessibility and affordability. While overall PBS expenditure for the government will likely remain high despite cost savings associated with generics, this may be offset by the introduction of new drugs onto the PBS Schedule. Based on the data presented in this study, predictive models for forecasting future cancer-related expenses will be critical for government budget planning across nations. In Australia, such models would provide comprehensive insights into the projected PBS costs for cancer drugs, which consistently constitute a significant portion of total cancer care costs each year (Australian Institute of Health and Welfare 2021).

### Limitations

There were some limitations in this analysis. Only cancer drugs that were listed on the PBS were included, as data on the use of private prescriptions could not be retrieved. Certain drugs, such as letrozole, methotrexate, and zoledronic acid, are also indicated for conditions beyond cancer treatment. Differentiating between services intended for cancer treatment and those for non-cancer conditions for the same drug was not feasible; therefore, the assumption was made that all services retrieved for dual-use drugs, provided the indication for cancer was PBS-subsidised, were attributed to cancer treatment. Although aflibercept was previously part of a discontinued protocol for treating advanced colorectal cancer in eviQ, it is currently exclusively subsidised for its ophthalmologic use in treating age-related macular degeneration under the brand name Eylea (Sarwar et al. 2016). Therefore, it was excluded from this study because all documented services from the PBS Item Reports are associated exclusively with ophthalmologic use. Data for 2023 were incomplete at the time of collection and was consequently not included for use. The “other agents” class included any drug whose mechanism of action was distinct from those in the other seven classes, resulting in a diverse list of drugs that ranged from carfilzomib, a proteasome inhibitor, to lenalidomide, a thalidomide analogue.

There are some inherent limitations of the PBS dataset, as it is influenced by changes in subsidy regulations, including approved indications and authority requirements, over time. In particular, medications dispensed at public hospitals prior to July 2013 may not have been completely documented, thereby influencing the precision of our PBS data collection during the early study years (2012–2013). The Public Hospital Pharmaceutical Reforms now enable participating public hospitals to dispense subsidised medications under agreements with other states and territories, but these agreements are subject to change (Mellish et al. 2015). Furthermore, PBS online data is sorted by the date of processing by the Department of Human Services rather than the date of dispensing, potentially causing delays, although this is less impactful for annual data, as was used for this analysis.

## Conclusions

At the time of this study, 141 drugs were listed on the PBS for the treatment of cancer, with services increasing at a higher rate than Australia’s population growth. While the most prescribed drugs are hormonal agents, of particular interest is the growing use of high-cost monoclonal antibody and kinase inhibitor drugs. Increased used of these drugs will subsequently put more pressure on governments worldwide to allocate sufficient funds in future budgeting cycles. Critical to this will be the development of accurate forecasting models that can take into account the rapidly changing patterns of use of the different drug classes for cancer.

## Supplementary Information

Below is the link to the electronic supplementary material.

Supplementary file1 (DOCX 50 KB)

## Data Availability

The data analysed for this study are available from Medicare Statistics at http://medicarestatistics.humanservices.gov.au/statistics/pbs_item.jsp.
