# Supplementary material for: An analysis of the trends in the usage of Pharmaceutical Benefits Scheme-subsidised cancer drugs in Australia from 2012 to 2022
Source: J Cancer Res Clin Oncol. 2024 Jul 31;150(8):375. doi: 10.1007/s00432-024-05889-x (PMC11291628; doi:10.1007/s00432-024-05889-x)
Supplement: Supplementary file 1 — Supplementary file1 (DOCX 50 KB) [file 432_2024_5889_MOESM1_ESM.docx]

PBS codes of drugs listed on the PBS Schedule and in eviQ “medical oncology” or “haematology” protocols

* denotes drugs included in superseded eviQ cancer treatment protocols

** denotes drugs included in discontinued eviQ cancer treatment protocols

**Green = only YTD (2023) data available**

**Red = no data available**

**Blue = excluded due to non-cancer indication**

Abemaciclib

[11876C](https://www.pbs.gov.au/medicine/item/11876c) - 50mg, 56

[11871T](https://www.pbs.gov.au/medicine/item/11871t) - 100mg, 56

[11868P](https://www.pbs.gov.au/medicine/item/11868p) - 150mg, 56

Abiraterone

[11206T](https://www.pbs.gov.au/medicine/item/11206t) - 500mg, 60

[2698B](https://www.pbs.gov.au/medicine/item/2698b) - 250mg, 120

[Abiraterone (micronised) & methylprednisolone](https://www.eviq.org.au/medical-oncology/urogenital/prostate/4355-prostate-metastatic-castration-resistant-abir)

[**13263C**](https://www.pbs.gov.au/medicine/item/13263c) - 125mcg, 120 & 4mg, 60

Acalabrutinib

[12117R](https://www.pbs.gov.au/medicine/item/12117r), [12826C](https://www.pbs.gov.au/medicine/item/12826c) - 100mg cap, 56

[**13318Y**](https://www.pbs.gov.au/medicine/item/13318y)**,** [**13325H**](https://www.pbs.gov.au/medicine/item/13325h) - 100mg tab, 56

Afatinib

[11335N](https://www.pbs.gov.au/medicine/item/11335n), [11336P](https://www.pbs.gov.au/medicine/item/11336p) - 20mg, 28

[11341X](https://www.pbs.gov.au/medicine/item/11341x), [11348G](https://www.pbs.gov.au/medicine/item/11348g) - 30mg

[11347F](https://www.pbs.gov.au/medicine/item/11347f), [11359W](https://www.pbs.gov.au/medicine/item/11359w) - 40mg

[11329G](https://www.pbs.gov.au/medicine/item/11329g), [11342Y](https://www.pbs.gov.au/medicine/item/11342y) - 50mg

Alectinib

[11226W](https://www.pbs.gov.au/medicine/item/11226w) - 150mg, 4x56

Anastrozole

[8179L](https://www.pbs.gov.au/medicine/item/8179l) - 1mg, 30

Apalutamide

[12992T](https://www.pbs.gov.au/medicine/item/12992t), [**13288J**](https://www.pbs.gov.au/medicine/item/13288j) - 60mg, 120

Arsenic trioxide (ATO)

[10691Q](https://www.pbs.gov.au/medicine/item/10691q), [10699D](https://www.pbs.gov.au/medicine/item/10699d), [4371C](https://www.pbs.gov.au/medicine/item/4371c), [7241D](https://www.pbs.gov.au/medicine/item/7241d) - 10x10mL

Asciminib

[**13248G**](https://www.pbs.gov.au/medicine/item/13248g)**,** [**13268H**](https://www.pbs.gov.au/medicine/item/13268h) - 20mg, 60

[**13259W**](https://www.pbs.gov.au/medicine/item/13259w)**,** [**13260X**](https://www.pbs.gov.au/medicine/item/13260x)**,** [**13264D**](https://www.pbs.gov.au/medicine/item/13264d) - 40mg, 60

Atezolizumab

[11277M](https://www.pbs.gov.au/medicine/item/11277m), [11284X](https://www.pbs.gov.au/medicine/item/11284x), [11297N](https://www.pbs.gov.au/medicine/item/11297n), [11309F](https://www.pbs.gov.au/medicine/item/11309f), [11792P](https://www.pbs.gov.au/medicine/item/11792p), [11801D](https://www.pbs.gov.au/medicine/item/11801d), [11802E](https://www.pbs.gov.au/medicine/item/11802e), [11807K](https://www.pbs.gov.au/medicine/item/11807k), [11926Q](https://www.pbs.gov.au/medicine/item/11926q), [11927R](https://www.pbs.gov.au/medicine/item/11927r), [11928T](https://www.pbs.gov.au/medicine/item/11928t), [11929W](https://www.pbs.gov.au/medicine/item/11929w), [12155R](https://www.pbs.gov.au/medicine/item/12155r), [12167J](https://www.pbs.gov.au/medicine/item/12167j), [12168K](https://www.pbs.gov.au/medicine/item/12168k), [12171N](https://www.pbs.gov.au/medicine/item/12171n), [13172G](https://www.pbs.gov.au/medicine/item/13172g), [13174J](https://www.pbs.gov.au/medicine/item/13174j) - 1.2g/20mL

[11930X](https://www.pbs.gov.au/medicine/item/11930x), [11931Y](https://www.pbs.gov.au/medicine/item/11931y), [11940K](https://www.pbs.gov.au/medicine/item/11940k), [11957H](https://www.pbs.gov.au/medicine/item/11957h), [12076N](https://www.pbs.gov.au/medicine/item/12076n), [12078Q](https://www.pbs.gov.au/medicine/item/12078q), [12097Q](https://www.pbs.gov.au/medicine/item/12097q), [12098R](https://www.pbs.gov.au/medicine/item/12098r), [12159Y](https://www.pbs.gov.au/medicine/item/12159y), [12174R](https://www.pbs.gov.au/medicine/item/12174r), [13170E](https://www.pbs.gov.au/medicine/item/13170e), [**13173H**](https://www.pbs.gov.au/medicine/item/13173h) - 840mg/14mL

Avelumab

[11671G](https://www.pbs.gov.au/medicine/item/11671g), [11679Q](https://www.pbs.gov.au/medicine/item/11679q), [11685B](https://www.pbs.gov.au/medicine/item/11685b), [11695M](https://www.pbs.gov.au/medicine/item/11695m), [13122P](https://www.pbs.gov.au/medicine/item/13122p), [13123Q](https://www.pbs.gov.au/medicine/item/13123q), [13126W](https://www.pbs.gov.au/medicine/item/13126w), [13132E](https://www.pbs.gov.au/medicine/item/13132e) - 200mg/10mL

Axitinib

[10539Q](https://www.pbs.gov.au/medicine/item/10539q), [10572K](https://www.pbs.gov.au/medicine/item/10572k) - 1mg, 28

[10540R](https://www.pbs.gov.au/medicine/item/10540r), [10556N](https://www.pbs.gov.au/medicine/item/10556n) - 5mg, 28

Azacitidine

[**13623B**](https://www.pbs.gov.au/medicine/item/13623b) - 200mg, 7

[**13619T**](https://www.pbs.gov.au/medicine/item/13619t)**,** [**13624C**](https://www.pbs.gov.au/medicine/item/13624c) - 300mg, 7

[12771E](https://www.pbs.gov.au/medicine/item/12771e), [12784W](https://www.pbs.gov.au/medicine/item/12784w), [13028Q](https://www.pbs.gov.au/medicine/item/13028q), [13033Y](https://www.pbs.gov.au/medicine/item/13033y), [13036D](https://www.pbs.gov.au/medicine/item/13036d), [13038F](https://www.pbs.gov.au/medicine/item/13038f), [13039G](https://www.pbs.gov.au/medicine/item/13039g), [13040H](https://www.pbs.gov.au/medicine/item/13040h), [13042K](https://www.pbs.gov.au/medicine/item/13042k), [13044M](https://www.pbs.gov.au/medicine/item/13044m), [6100C](https://www.pbs.gov.au/medicine/item/6100c), [6138C](https://www.pbs.gov.au/medicine/item/6138c), [9597D](https://www.pbs.gov.au/medicine/item/9597d), [9598E](https://www.pbs.gov.au/medicine/item/9598e) - 100mg injection

Bendamustine

[10760H](https://www.pbs.gov.au/medicine/item/10760h), [10763L](https://www.pbs.gov.au/medicine/item/10763l) - 25mg, 100mg injections

Bevacizumab

[12479T](https://www.pbs.gov.au/medicine/item/12479t), [12508H](https://www.pbs.gov.au/medicine/item/12508h) - 100mg/4mL, 400mg/16mL injections

Bicalutamide

[8094B](https://www.pbs.gov.au/medicine/item/8094b) - 50mg, 28

Binimetinib

[11948W](https://www.pbs.gov.au/medicine/item/11948w), [11961M](https://www.pbs.gov.au/medicine/item/11961m) - 15mg, 84

Bleomycin

[4433H](https://www.pbs.gov.au/medicine/item/4433h), [7244G](https://www.pbs.gov.au/medicine/item/7244g) - 15000IU injection

Blinatumomab

[11115B](https://www.pbs.gov.au/medicine/item/11115b), [11116C](https://www.pbs.gov.au/medicine/item/11116c), [11117D](https://www.pbs.gov.au/medicine/item/11117d), [11118E](https://www.pbs.gov.au/medicine/item/11118e), [11119F](https://www.pbs.gov.au/medicine/item/11119f), [11120G](https://www.pbs.gov.au/medicine/item/11120g), [11850Q](https://www.pbs.gov.au/medicine/item/11850q), [11867N](https://www.pbs.gov.au/medicine/item/11867n) - 38.5mcg injection

Bortezomib

[12219D](https://www.pbs.gov.au/medicine/item/12219d), [12227M](https://www.pbs.gov.au/medicine/item/12227m) - 3mg, 2.5mg, 1mg, 3.5mg/1.4mL, 2.5mg/mL, 3.5mg injections

Brentuximab vedotin

[10166C](https://www.pbs.gov.au/medicine/item/10166c), [10171H](https://www.pbs.gov.au/medicine/item/10171h), [10172J](https://www.pbs.gov.au/medicine/item/10172j), [10180T](https://www.pbs.gov.au/medicine/item/10180t), [11067L](https://www.pbs.gov.au/medicine/item/11067l), [11073T](https://www.pbs.gov.au/medicine/item/11073t), [11079D](https://www.pbs.gov.au/medicine/item/11079d), [11080E](https://www.pbs.gov.au/medicine/item/11080e), [11086L](https://www.pbs.gov.au/medicine/item/11086l), [11087M](https://www.pbs.gov.au/medicine/item/11087m), [11089P](https://www.pbs.gov.au/medicine/item/11089p), [11096B](https://www.pbs.gov.au/medicine/item/11096b), [11651F](https://www.pbs.gov.au/medicine/item/11651f), [11660Q](https://www.pbs.gov.au/medicine/item/11660q), [11661R](https://www.pbs.gov.au/medicine/item/11661r), [11664X](https://www.pbs.gov.au/medicine/item/11664x), [12632W](https://www.pbs.gov.au/medicine/item/12632w), [12646N](https://www.pbs.gov.au/medicine/item/12646n), [12656D](https://www.pbs.gov.au/medicine/item/12656d), [12657E](https://www.pbs.gov.au/medicine/item/12657e) - 50mg injection

Brigatinib

[11984R](https://www.pbs.gov.au/medicine/item/11984r) - 180mg, 28

[11974F](https://www.pbs.gov.au/medicine/item/11974f) - 90mg, 28

[11980M](https://www.pbs.gov.au/medicine/item/11980m) - 30mg, 28

[11976H](https://www.pbs.gov.au/medicine/item/11976h) - 90mgx7, 180mgx21

Cabazitaxel

[4376H](https://www.pbs.gov.au/medicine/item/4376h), [7236W](https://www.pbs.gov.au/medicine/item/7236w) - 60mg/6mL, 60mg/1.5mL, 60mg/3mL injections

Cabozantinib

[11371L](https://www.pbs.gov.au/medicine/item/11371l), [11374P](https://www.pbs.gov.au/medicine/item/11374p) - 20mg, 30

[11368H](https://www.pbs.gov.au/medicine/item/11368h), [11369J](https://www.pbs.gov.au/medicine/item/11369j) - 40mg, 30

[11360X](https://www.pbs.gov.au/medicine/item/11360x), [11367G](https://www.pbs.gov.au/medicine/item/11367g) - 60mg, 30

Capecitabine

[8361C](https://www.pbs.gov.au/medicine/item/8361c) - 150mg, 60

[8362D](https://www.pbs.gov.au/medicine/item/8362d) - 500mg, 120

Carboplatin

[4309T](https://www.pbs.gov.au/medicine/item/4309t), [7222D](https://www.pbs.gov.au/medicine/item/7222d) - 450mg/45mL injection

Carfilzomib

[11229B](https://www.pbs.gov.au/medicine/item/11229b), [11230C](https://www.pbs.gov.au/medicine/item/11230c), [12243J](https://www.pbs.gov.au/medicine/item/12243j), [12244K](https://www.pbs.gov.au/medicine/item/12244k), [**13637R**](https://www.pbs.gov.au/medicine/item/13637r)**,** [**13638T**](https://www.pbs.gov.au/medicine/item/13638t) - 10mg, 30mg, 60mg injections

Carmustine

[8898H](https://www.pbs.gov.au/medicine/item/8898h) - 7.7mg implant, x8

Cemiplimab

[13135H](https://www.pbs.gov.au/medicine/item/13135h), [13152F](https://www.pbs.gov.au/medicine/item/13152f), [13153G](https://www.pbs.gov.au/medicine/item/13153g), [13159N](https://www.pbs.gov.au/medicine/item/13159n), [**13160P**](https://www.pbs.gov.au/medicine/item/13160p)**,** [13161Q](https://www.pbs.gov.au/medicine/item/13161q), [**13162R**](https://www.pbs.gov.au/medicine/item/13162r)**,** [**13169D**](https://www.pbs.gov.au/medicine/item/13169d)

- 350mg/7mL injection

Cetuximab

[10262D](https://www.pbs.gov.au/medicine/item/10262d), [10265G](https://www.pbs.gov.au/medicine/item/10265g), [12816M](https://www.pbs.gov.au/medicine/item/12816m), [12817N](https://www.pbs.gov.au/medicine/item/12817n), [12820R](https://www.pbs.gov.au/medicine/item/12820r), [12821T](https://www.pbs.gov.au/medicine/item/12821t), [4312Y](https://www.pbs.gov.au/medicine/item/4312y), [4435K](https://www.pbs.gov.au/medicine/item/4435k), [4436L](https://www.pbs.gov.au/medicine/item/4436l), [4731B](https://www.pbs.gov.au/medicine/item/4731b), [7223E](https://www.pbs.gov.au/medicine/item/7223e), [7240C](https://www.pbs.gov.au/medicine/item/7240c), [7242E](https://www.pbs.gov.au/medicine/item/7242e), [7273T](https://www.pbs.gov.au/medicine/item/7273t) - 100mg/20mL, 500mg/100mL injections

Chlorambucil

[1163F](https://www.pbs.gov.au/medicine/item/1163f) - 2mg, 25

Cisplatin

[4319H](https://www.pbs.gov.au/medicine/item/4319h), [7224F](https://www.pbs.gov.au/medicine/item/7224f) - 100mg/100mL, 50mg/50mL

Cladribine

[11603Q](https://www.pbs.gov.au/medicine/item/11603q) - 10mg, 1

[11604R](https://www.pbs.gov.au/medicine/item/11604r) - 10mg, 4

[11611D](https://www.pbs.gov.au/medicine/item/11611d) - 10mg, 6

[4326Q](https://www.pbs.gov.au/medicine/item/4326q), [7225G](https://www.pbs.gov.au/medicine/item/7225g) - 10mg/5mL, 10mg/10mL

Cobimetinib

[11074W](https://www.pbs.gov.au/medicine/item/11074w), [11075X](https://www.pbs.gov.au/medicine/item/11075x) - 20mg, 63

Cyclophosphamide

[1266P](https://www.pbs.gov.au/medicine/item/1266p) - 50mg, 50

[4327R](https://www.pbs.gov.au/medicine/item/4327r), [7226H](https://www.pbs.gov.au/medicine/item/7226h) - 2g, 500mg, 1g injections

Cyproterone

[8019C](https://www.pbs.gov.au/medicine/item/8019c) - 100mg, 50

[1269T](https://www.pbs.gov.au/medicine/item/1269t) - 50mg, 20

[1270W](https://www.pbs.gov.au/medicine/item/1270w) - 50mg, 50

Cytarabine

[4357H](https://www.pbs.gov.au/medicine/item/4357h), [7227J](https://www.pbs.gov.au/medicine/item/7227j) - 100mg/5mL x 5

Dabrafenib

[10003L](https://www.pbs.gov.au/medicine/item/10003l), [11823G](https://www.pbs.gov.au/medicine/item/11823g), [2846T](https://www.pbs.gov.au/medicine/item/2846t) - 75mg, 120

[11820D](https://www.pbs.gov.au/medicine/item/11820d), [2954L](https://www.pbs.gov.au/medicine/item/2954l), [2963Y](https://www.pbs.gov.au/medicine/item/2963y) - 50mg

Daratumumab

[12673B](https://www.pbs.gov.au/medicine/item/12673b), [12682L](https://www.pbs.gov.au/medicine/item/12682l), [12683M](https://www.pbs.gov.au/medicine/item/12683m), [12704P](https://www.pbs.gov.au/medicine/item/12704p), [12725R](https://www.pbs.gov.au/medicine/item/12725r), [12745T](https://www.pbs.gov.au/medicine/item/12745t), [12746W](https://www.pbs.gov.au/medicine/item/12746w), [12755H](https://www.pbs.gov.au/medicine/item/12755h), [**13199Q**](https://www.pbs.gov.au/medicine/item/13199q)**,** [**13201T**](https://www.pbs.gov.au/medicine/item/13201t)**,** [**13202W**](https://www.pbs.gov.au/medicine/item/13202w)**,** [**13203X**](https://www.pbs.gov.au/medicine/item/13203x) - 1.8g/15mL

[12220E](https://www.pbs.gov.au/medicine/item/12220e), [12221F](https://www.pbs.gov.au/medicine/item/12221f), [12225K](https://www.pbs.gov.au/medicine/item/12225k), [12226L](https://www.pbs.gov.au/medicine/item/12226l), [12228N](https://www.pbs.gov.au/medicine/item/12228n), [12229P](https://www.pbs.gov.au/medicine/item/12229p), [12230Q](https://www.pbs.gov.au/medicine/item/12230q), [12231R](https://www.pbs.gov.au/medicine/item/12231r) - 100mg/5mL, 400mg/20mL

Darolutamide

[12684N](https://www.pbs.gov.au/medicine/item/12684n) - 300mg, 112

Dasatinib

[12843Y](https://www.pbs.gov.au/medicine/item/12843y), [12857Q](https://www.pbs.gov.au/medicine/item/12857q), [12860W](https://www.pbs.gov.au/medicine/item/12860w), [12865D](https://www.pbs.gov.au/medicine/item/12865d), [1381Q](https://www.pbs.gov.au/medicine/item/1381q), [2482P](https://www.pbs.gov.au/medicine/item/2482p), [9126H](https://www.pbs.gov.au/medicine/item/9126h) - 50mg, 60

[12866E](https://www.pbs.gov.au/medicine/item/12866e), [12886F](https://www.pbs.gov.au/medicine/item/12886f), [12890K](https://www.pbs.gov.au/medicine/item/12890k), [12903D](https://www.pbs.gov.au/medicine/item/12903d), [1415L](https://www.pbs.gov.au/medicine/item/1415l), [2485T](https://www.pbs.gov.au/medicine/item/2485t), [9127J](https://www.pbs.gov.au/medicine/item/9127j) - 70mg, 60

[12842X](https://www.pbs.gov.au/medicine/item/12842x), [12859T](https://www.pbs.gov.au/medicine/item/12859t), [12889J](https://www.pbs.gov.au/medicine/item/12889j), [12902C](https://www.pbs.gov.au/medicine/item/12902c), [1416M](https://www.pbs.gov.au/medicine/item/1416m), [9342Q](https://www.pbs.gov.au/medicine/item/9342q), [9343R](https://www.pbs.gov.au/medicine/item/9343r) - 100mg, 30

[12849G](https://www.pbs.gov.au/medicine/item/12849g), [12850H](https://www.pbs.gov.au/medicine/item/12850h), [12869H](https://www.pbs.gov.au/medicine/item/12869h), [12888H](https://www.pbs.gov.au/medicine/item/12888h), [1354G](https://www.pbs.gov.au/medicine/item/1354g), [2478K](https://www.pbs.gov.au/medicine/item/2478k), [9125G](https://www.pbs.gov.au/medicine/item/9125g) - 20mg, 60

Degarelix

[2784M](https://www.pbs.gov.au/medicine/item/2784m) - 80mg injection

[2785N](https://www.pbs.gov.au/medicine/item/2785n) - 120mg injection x2

Denosumab

[10061M](https://www.pbs.gov.au/medicine/item/10061m), [5110Y](https://www.pbs.gov.au/medicine/item/5110y) - 120mg/1.7mL

[**5457F**](https://www.pbs.gov.au/medicine/item/5457f) - 60mg/mL injection

Dexamethasone

[2507Y](https://www.pbs.gov.au/medicine/item/2507y) - 4mg, 30

[1292B](https://www.pbs.gov.au/medicine/item/1292b) - 500mcg, 30

[**1288T**](https://www.pbs.gov.au/medicine/item/1288t)**,** [**5565X**](https://www.pbs.gov.au/medicine/item/5565x) - 0.1% eye drops

Docetaxel

[10148D](https://www.pbs.gov.au/medicine/item/10148d), [10158P](https://www.pbs.gov.au/medicine/item/10158p) - 160mg/8mL, 160mg/16mL, 80mg/4mL, 80mg/8mL

Doxorubicin

[4361M](https://www.pbs.gov.au/medicine/item/4361m), [7229L](https://www.pbs.gov.au/medicine/item/7229l) - 200mg/100mL, 50mg/25mL

Doxorubicin liposomal

[4364Q](https://www.pbs.gov.au/medicine/item/4364q), [5705G](https://www.pbs.gov.au/medicine/item/5705g), [6249X](https://www.pbs.gov.au/medicine/item/6249x), [7230M](https://www.pbs.gov.au/medicine/item/7230m) - 20mg/10mL, 50mg/25mL

Durvalumab

[11911X](https://www.pbs.gov.au/medicine/item/11911x), [11915D](https://www.pbs.gov.au/medicine/item/11915d) - 120mg/2.4mL, 500mg/10mL

Elotuzumab

[12983H](https://www.pbs.gov.au/medicine/item/12983h), [12989P](https://www.pbs.gov.au/medicine/item/12989p), [12990Q](https://www.pbs.gov.au/medicine/item/12990q), [12995Y](https://www.pbs.gov.au/medicine/item/12995y) - 300mg, 400mg injections

Encorafenib

[11937G](https://www.pbs.gov.au/medicine/item/11937g), [11954E](https://www.pbs.gov.au/medicine/item/11954e) - 50mg, 28

[11938H](https://www.pbs.gov.au/medicine/item/11938h), [11949X](https://www.pbs.gov.au/medicine/item/11949x), [12814K](https://www.pbs.gov.au/medicine/item/12814k), [12815L](https://www.pbs.gov.au/medicine/item/12815l) - 75mg, 42

Enfortumab vedotin

[**13634N**](https://www.pbs.gov.au/medicine/item/13634n)**,** [**13648H**](https://www.pbs.gov.au/medicine/item/13648h) - 20mg, 30mg injections

Entrectinib

[12092K](https://www.pbs.gov.au/medicine/item/12092k) - 200mg, 90

Enzalutamide

[10174L](https://www.pbs.gov.au/medicine/item/10174l), [13118K](https://www.pbs.gov.au/medicine/item/13118k), [**13353T**](https://www.pbs.gov.au/medicine/item/13353t) - 40mg, 112

Epirubicin

[4375G](https://www.pbs.gov.au/medicine/item/4375g), [7231N](https://www.pbs.gov.au/medicine/item/7231n) - 200mg/100mL

Eribulin

[10140Q](https://www.pbs.gov.au/medicine/item/10140q), [10144X](https://www.pbs.gov.au/medicine/item/10144x), [11199K](https://www.pbs.gov.au/medicine/item/11199k), [11212D](https://www.pbs.gov.au/medicine/item/11212d) - 1mg/2mL

Etoposide

[4428C](https://www.pbs.gov.au/medicine/item/4428c), [7237X](https://www.pbs.gov.au/medicine/item/7237x) - 1gx1, 100mg/5mLx5 injections

Everolimus

[11591C](https://www.pbs.gov.au/medicine/item/11591c), [11607X](https://www.pbs.gov.au/medicine/item/11607x) - 2mg disp, 30

[11599L](https://www.pbs.gov.au/medicine/item/11599l), [11608Y](https://www.pbs.gov.au/medicine/item/11608y) - 3mg disp, 30

[11592D](https://www.pbs.gov.au/medicine/item/11592d), [11598K](https://www.pbs.gov.au/medicine/item/11598k) - 5mg disp, 30

[11258M](https://www.pbs.gov.au/medicine/item/11258m), [2818H](https://www.pbs.gov.au/medicine/item/2818h) - 2.5mg, 30

[10132G](https://www.pbs.gov.au/medicine/item/10132g), [10135K](https://www.pbs.gov.au/medicine/item/10135k), [11262R](https://www.pbs.gov.au/medicine/item/11262r), [11267B](https://www.pbs.gov.au/medicine/item/11267b), [11377T](https://www.pbs.gov.au/medicine/item/11377t), [2985D](https://www.pbs.gov.au/medicine/item/2985d) - 10mg, 30

[10131F](https://www.pbs.gov.au/medicine/item/10131f), [10133H](https://www.pbs.gov.au/medicine/item/10133h), [11254H](https://www.pbs.gov.au/medicine/item/11254h), [11257L](https://www.pbs.gov.au/medicine/item/11257l), [11362B](https://www.pbs.gov.au/medicine/item/11362b), [2819J](https://www.pbs.gov.au/medicine/item/2819j) - 5mg, 30

[5738B](https://www.pbs.gov.au/medicine/item/5738b), [6459Y](https://www.pbs.gov.au/medicine/item/6459y), [8840G](https://www.pbs.gov.au/medicine/item/8840g) - 250mcg, 60

[5739C](https://www.pbs.gov.au/medicine/item/5739c), [6460B](https://www.pbs.gov.au/medicine/item/6460b), [8841H](https://www.pbs.gov.au/medicine/item/8841h) - 500mcg, 60

[5740D](https://www.pbs.gov.au/medicine/item/5740d), [6461C](https://www.pbs.gov.au/medicine/item/6461c), [8842J](https://www.pbs.gov.au/medicine/item/8842j) - 750mcg, 60

[5737Y](https://www.pbs.gov.au/medicine/item/5737y), [9352F](https://www.pbs.gov.au/medicine/item/9352f), [9582H](https://www.pbs.gov.au/medicine/item/9582h) - 1mg, 60

Exemestane

[10103R](https://www.pbs.gov.au/medicine/item/10103r), [8506Q](https://www.pbs.gov.au/medicine/item/8506q) - 25mg, 30

Fludarabine

[9184J](https://www.pbs.gov.au/medicine/item/9184j) - 10mg, 20

[4393F](https://www.pbs.gov.au/medicine/item/4393f), [7233Q](https://www.pbs.gov.au/medicine/item/7233q) - 50mg, 50mg/2mL injections

Fluorouracil

[4394G](https://www.pbs.gov.au/medicine/item/4394g), [4431F](https://www.pbs.gov.au/medicine/item/4431f), [7234R](https://www.pbs.gov.au/medicine/item/7234r), [7239B](https://www.pbs.gov.au/medicine/item/7239b) - 1g/20mL, 2.5g/50mL, 500mg/10mL, 5g/100mL IV injections

[**4222F**](https://www.pbs.gov.au/medicine/item/4222f) - 5% cream

[**13758D**](https://www.pbs.gov.au/medicine/item/13758d) - 4% cream

Fulvestrant

[12300J](https://www.pbs.gov.au/medicine/item/12300j) - 250mg/5mLx2

Gemcitabine

[4439P](https://www.pbs.gov.au/medicine/item/4439p), [7246J](https://www.pbs.gov.au/medicine/item/7246j) - 1g/26.3mL, 2g/52.6mL

Gemtuzumab ozogamicin

[**12844B**](https://www.pbs.gov.au/medicine/item/12844b), [12861X](https://www.pbs.gov.au/medicine/item/12861x), [12878T](https://www.pbs.gov.au/medicine/item/12878t), [12904E](https://www.pbs.gov.au/medicine/item/12904e) - 5mg injection

Gilteritinib

[13093D](https://www.pbs.gov.au/medicine/item/13093d), [13094E](https://www.pbs.gov.au/medicine/item/13094e) - 40mg, 84

Goserelin

[8093Y](https://www.pbs.gov.au/medicine/item/8093y) - 10.8mg implant

[1454M](https://www.pbs.gov.au/medicine/item/1454m) - 3.6mg implant

Goserelin & bicalutamide combined

[9065D](https://www.pbs.gov.au/medicine/item/9065d) - 10.8/50mg, 28

[9066E](https://www.pbs.gov.au/medicine/item/9066e) - 10.8/50mg, 84

[9064C](https://www.pbs.gov.au/medicine/item/9064c) - 3.6/5mg, 28

Ibrutinib

[11213E](https://www.pbs.gov.au/medicine/item/11213e) - 140mg, 90

[11419B](https://www.pbs.gov.au/medicine/item/11419b) - 140mg, 120

Idarubicin

[4440Q](https://www.pbs.gov.au/medicine/item/4440q), [7247K](https://www.pbs.gov.au/medicine/item/7247k) - 5mg/5mL

Idelalisib

[11170X](https://www.pbs.gov.au/medicine/item/11170x), [11171Y](https://www.pbs.gov.au/medicine/item/11171y), [12813J](https://www.pbs.gov.au/medicine/item/12813j) - 100mg, 60

[11162L](https://www.pbs.gov.au/medicine/item/11162l), [11165P](https://www.pbs.gov.au/medicine/item/11165p), [12812H](https://www.pbs.gov.au/medicine/item/12812h) - 150mg

Ifosfamide

[4448D](https://www.pbs.gov.au/medicine/item/4448d), [7248L](https://www.pbs.gov.au/medicine/item/7248l) - 1g, 2g injections

Imatinib

[10915L](https://www.pbs.gov.au/medicine/item/10915l), [10918P](https://www.pbs.gov.au/medicine/item/10918p), [10920R](https://www.pbs.gov.au/medicine/item/10920r), [10924Y](https://www.pbs.gov.au/medicine/item/10924y), [10940T](https://www.pbs.gov.au/medicine/item/10940t), [10941W](https://www.pbs.gov.au/medicine/item/10941w), [10942X](https://www.pbs.gov.au/medicine/item/10942x), [11757T](https://www.pbs.gov.au/medicine/item/11757t), [11770L](https://www.pbs.gov.au/medicine/item/11770l), [11776T](https://www.pbs.gov.au/medicine/item/11776t), [11777W](https://www.pbs.gov.au/medicine/item/11777w), [11782D](https://www.pbs.gov.au/medicine/item/11782d), [11783E](https://www.pbs.gov.au/medicine/item/11783e), [11875B](https://www.pbs.gov.au/medicine/item/11875b), [12709X](https://www.pbs.gov.au/medicine/item/12709x), [12710Y](https://www.pbs.gov.au/medicine/item/12710y), [12722N](https://www.pbs.gov.au/medicine/item/12722n), [12759M](https://www.pbs.gov.au/medicine/item/12759m) - 100mg cap, 60

[11753N](https://www.pbs.gov.au/medicine/item/11753n), [11762C](https://www.pbs.gov.au/medicine/item/11762c), [11769K](https://www.pbs.gov.au/medicine/item/11769k), [11775R](https://www.pbs.gov.au/medicine/item/11775r), [11780B](https://www.pbs.gov.au/medicine/item/11780b), [11781C](https://www.pbs.gov.au/medicine/item/11781c), [11784F](https://www.pbs.gov.au/medicine/item/11784f), [11787J](https://www.pbs.gov.au/medicine/item/11787j), [11880G](https://www.pbs.gov.au/medicine/item/11880g), [5443L](https://www.pbs.gov.au/medicine/item/5443l), [9111M](https://www.pbs.gov.au/medicine/item/9111m), [9113P](https://www.pbs.gov.au/medicine/item/9113p), [9115R](https://www.pbs.gov.au/medicine/item/9115r), [9123E](https://www.pbs.gov.au/medicine/item/9123e), [9172R](https://www.pbs.gov.au/medicine/item/9172r), [9174W](https://www.pbs.gov.au/medicine/item/9174w), [9176Y](https://www.pbs.gov.au/medicine/item/9176y), [9178C](https://www.pbs.gov.au/medicine/item/9178c) - 100mg tab, 60

[10916M](https://www.pbs.gov.au/medicine/item/10916m), [10917N](https://www.pbs.gov.au/medicine/item/10917n), [10921T](https://www.pbs.gov.au/medicine/item/10921t), [10925B](https://www.pbs.gov.au/medicine/item/10925b), [10933K](https://www.pbs.gov.au/medicine/item/10933k), [10935M](https://www.pbs.gov.au/medicine/item/10935m), [10939R](https://www.pbs.gov.au/medicine/item/10939r), [11756R](https://www.pbs.gov.au/medicine/item/11756r), [11763D](https://www.pbs.gov.au/medicine/item/11763d), [11764E](https://www.pbs.gov.au/medicine/item/11764e), [11771M](https://www.pbs.gov.au/medicine/item/11771m), [11772N](https://www.pbs.gov.au/medicine/item/11772n), [11779Y](https://www.pbs.gov.au/medicine/item/11779y), [11870R](https://www.pbs.gov.au/medicine/item/11870r), [12681K](https://www.pbs.gov.au/medicine/item/12681k), [12711B](https://www.pbs.gov.au/medicine/item/12711b), [12723P](https://www.pbs.gov.au/medicine/item/12723p), [12754G](https://www.pbs.gov.au/medicine/item/12754g) - 400mg cap, 30

[11752M](https://www.pbs.gov.au/medicine/item/11752m), [11758W](https://www.pbs.gov.au/medicine/item/11758w), [11765F](https://www.pbs.gov.au/medicine/item/11765f), [11778X](https://www.pbs.gov.au/medicine/item/11778x), [11785G](https://www.pbs.gov.au/medicine/item/11785g), [11786H](https://www.pbs.gov.au/medicine/item/11786h), [11788K](https://www.pbs.gov.au/medicine/item/11788k), [11789L](https://www.pbs.gov.au/medicine/item/11789l), [11878E](https://www.pbs.gov.au/medicine/item/11878e), [5444M](https://www.pbs.gov.au/medicine/item/5444m), [9112N](https://www.pbs.gov.au/medicine/item/9112n), [9114Q](https://www.pbs.gov.au/medicine/item/9114q), [9116T](https://www.pbs.gov.au/medicine/item/9116t), [9124F](https://www.pbs.gov.au/medicine/item/9124f), [9173T](https://www.pbs.gov.au/medicine/item/9173t), [9175X](https://www.pbs.gov.au/medicine/item/9175x), [9177B](https://www.pbs.gov.au/medicine/item/9177b), [9179D](https://www.pbs.gov.au/medicine/item/9179d) - 400mg tab, 30

[**12911M**](https://www.pbs.gov.au/medicine/item/12911m)**,** [12912N](https://www.pbs.gov.au/medicine/item/12912n), [12919Y](https://www.pbs.gov.au/medicine/item/12919y), [**12920B**](https://www.pbs.gov.au/medicine/item/12920b)**,** [**12923E**](https://www.pbs.gov.au/medicine/item/12923e)**,** [**12924F**](https://www.pbs.gov.au/medicine/item/12924f)**,** [**12926H**](https://www.pbs.gov.au/medicine/item/12926h)**,** [**12927J**](https://www.pbs.gov.au/medicine/item/12927j)**,** [**12928K**](https://www.pbs.gov.au/medicine/item/12928k)**,** [**12935T**](https://www.pbs.gov.au/medicine/item/12935t) - 600mg tab, 30

Inotuzumab ozogamicin

[11668D](https://www.pbs.gov.au/medicine/item/11668d), [11673J](https://www.pbs.gov.au/medicine/item/11673j), [11680R](https://www.pbs.gov.au/medicine/item/11680r), [11696N](https://www.pbs.gov.au/medicine/item/11696n) - 1mg injection

Ipilimumab

[11628B](https://www.pbs.gov.au/medicine/item/11628b), [11644W](https://www.pbs.gov.au/medicine/item/11644w), [12304N](https://www.pbs.gov.au/medicine/item/12304n), [12322M](https://www.pbs.gov.au/medicine/item/12322m), [12583G](https://www.pbs.gov.au/medicine/item/12583g), [12601F](https://www.pbs.gov.au/medicine/item/12601f), [2638W](https://www.pbs.gov.au/medicine/item/2638w), [2641B](https://www.pbs.gov.au/medicine/item/2641b) - 50mg/10mL

[2638W](https://www.pbs.gov.au/medicine/item/2638w), [2641B](https://www.pbs.gov.au/medicine/item/2641b) - 50mg/10ml and 200mg/40mL

Irinotecan

[4451G](https://www.pbs.gov.au/medicine/item/4451g), [7249M](https://www.pbs.gov.au/medicine/item/7249m) - 100mg/5mL, 40mg/2mL, 500mg/25mL

Lanreotide (Somatuline autogel)

[11289E](https://www.pbs.gov.au/medicine/item/11289e), [11513Y](https://www.pbs.gov.au/medicine/item/11513y), [11527Q](https://www.pbs.gov.au/medicine/item/11527q), [11736Q](https://www.pbs.gov.au/medicine/item/11736q), [5779E](https://www.pbs.gov.au/medicine/item/5779e), [6425E](https://www.pbs.gov.au/medicine/item/6425e) - 120mg/0.5mL

[11315M](https://www.pbs.gov.au/medicine/item/11315m), [5777C](https://www.pbs.gov.au/medicine/item/5777c), [6423C](https://www.pbs.gov.au/medicine/item/6423c) - 60mg/0.5mL

[11316N](https://www.pbs.gov.au/medicine/item/11316n), [5778D](https://www.pbs.gov.au/medicine/item/5778d), [6424D](https://www.pbs.gov.au/medicine/item/6424d) - 90mg/0.5mL

Lapatinib

[11251E](https://www.pbs.gov.au/medicine/item/11251e), [9148L](https://www.pbs.gov.au/medicine/item/9148l) - 250mg, 70

Lenalidomide

[12035K](https://www.pbs.gov.au/medicine/item/12035k), [12058P](https://www.pbs.gov.au/medicine/item/12058p) - 5mg, 14

[11029L](https://www.pbs.gov.au/medicine/item/11029l), [11036W](https://www.pbs.gov.au/medicine/item/11036w), [12034J](https://www.pbs.gov.au/medicine/item/12034j), [12038N](https://www.pbs.gov.au/medicine/item/12038n), [12039P](https://www.pbs.gov.au/medicine/item/12039p), [12071H](https://www.pbs.gov.au/medicine/item/12071h), [12984J](https://www.pbs.gov.au/medicine/item/12984j), [12985K](https://www.pbs.gov.au/medicine/item/12985k), [**13636Q**](https://www.pbs.gov.au/medicine/item/13636q)**,** [**13642B**](https://www.pbs.gov.au/medicine/item/13642b)**,** [2798G](https://www.pbs.gov.au/medicine/item/2798g), [2799H](https://www.pbs.gov.au/medicine/item/2799h), [5783J](https://www.pbs.gov.au/medicine/item/5783j), [9642L](https://www.pbs.gov.au/medicine/item/9642l) - 5mg, 21

[11966T](https://www.pbs.gov.au/medicine/item/11966t), [11967W](https://www.pbs.gov.au/medicine/item/11967w) - 5mg, 28

[12004T](https://www.pbs.gov.au/medicine/item/12004t), [12070G](https://www.pbs.gov.au/medicine/item/12070g) - 10mg, 14

[11063G](https://www.pbs.gov.au/medicine/item/11063g), [11064H](https://www.pbs.gov.au/medicine/item/11064h), [12050F](https://www.pbs.gov.au/medicine/item/12050f), [12057N](https://www.pbs.gov.au/medicine/item/12057n), [12060R](https://www.pbs.gov.au/medicine/item/12060r), [12061T](https://www.pbs.gov.au/medicine/item/12061t), [12980E](https://www.pbs.gov.au/medicine/item/12980e), [12988N](https://www.pbs.gov.au/medicine/item/12988n), [**13658W**](https://www.pbs.gov.au/medicine/item/13658w)**,** [**13661B**](https://www.pbs.gov.au/medicine/item/13661b)**,** [2796E](https://www.pbs.gov.au/medicine/item/2796e), [2802L](https://www.pbs.gov.au/medicine/item/2802l), [5784K](https://www.pbs.gov.au/medicine/item/5784k), [9643M](https://www.pbs.gov.au/medicine/item/9643m) - 10mg, 21

[11968X](https://www.pbs.gov.au/medicine/item/11968x), [11969Y](https://www.pbs.gov.au/medicine/item/11969y) - 10mg, 28

[12012F](https://www.pbs.gov.au/medicine/item/12012f), [12069F](https://www.pbs.gov.au/medicine/item/12069f) - 15mg, 14

[11042E](https://www.pbs.gov.au/medicine/item/11042e), [11062F](https://www.pbs.gov.au/medicine/item/11062f), [12011E](https://www.pbs.gov.au/medicine/item/12011e), [12020P](https://www.pbs.gov.au/medicine/item/12020p), [12026Y](https://www.pbs.gov.au/medicine/item/12026y), [12062W](https://www.pbs.gov.au/medicine/item/12062w), [12986L](https://www.pbs.gov.au/medicine/item/12986l), [12991R](https://www.pbs.gov.au/medicine/item/12991r), [**13641Y**](https://www.pbs.gov.au/medicine/item/13641y)**,** [**13657T**](https://www.pbs.gov.au/medicine/item/13657t)**,** [5785L](https://www.pbs.gov.au/medicine/item/5785l), [9644N](https://www.pbs.gov.au/medicine/item/9644n) - 15mg, 21

[11964Q](https://www.pbs.gov.au/medicine/item/11964q), [11965R](https://www.pbs.gov.au/medicine/item/11965r) - 15mg, 28

[12018M](https://www.pbs.gov.au/medicine/item/12018m), [12019N](https://www.pbs.gov.au/medicine/item/12019n) - 25mg, 14

[11041D](https://www.pbs.gov.au/medicine/item/11041d), [11055W](https://www.pbs.gov.au/medicine/item/11055w), [12036L](https://www.pbs.gov.au/medicine/item/12036l), [12037M](https://www.pbs.gov.au/medicine/item/12037m), [12059Q](https://www.pbs.gov.au/medicine/item/12059q), [12068E](https://www.pbs.gov.au/medicine/item/12068e), [12979D](https://www.pbs.gov.au/medicine/item/12979d), [12993W](https://www.pbs.gov.au/medicine/item/12993w), [**13630J**](https://www.pbs.gov.au/medicine/item/13630j)**,** [**13660Y**](https://www.pbs.gov.au/medicine/item/13660y)**,** [5786M](https://www.pbs.gov.au/medicine/item/5786m), [9645P](https://www.pbs.gov.au/medicine/item/9645p) - 25mg, 21

Lenvatinib

[10965D](https://www.pbs.gov.au/medicine/item/10965d), [**13253M**](https://www.pbs.gov.au/medicine/item/13253m)**,** [**13283D**](https://www.pbs.gov.au/medicine/item/13283d) - 10mg, 30

[10952K](https://www.pbs.gov.au/medicine/item/10952k), [11638M](https://www.pbs.gov.au/medicine/item/11638m), [**13252L**](https://www.pbs.gov.au/medicine/item/13252l)**,** [**13290L**](https://www.pbs.gov.au/medicine/item/13290l) - 4mg, 30

Letrozole

[8245Y](https://www.pbs.gov.au/medicine/item/8245y) - 2.5mg, 30

Leuprorelin

[11943N](https://www.pbs.gov.au/medicine/item/11943n) - 45mg MR chamber

[8876E](https://www.pbs.gov.au/medicine/item/8876e) - 22.5mg

[11944P](https://www.pbs.gov.au/medicine/item/11944p) - 30mg

[8875D](https://www.pbs.gov.au/medicine/item/8875d) - 7.5mg

[8708H](https://www.pbs.gov.au/medicine/item/8708h) - 22.5mg MR syringe

[8709J](https://www.pbs.gov.au/medicine/item/8709j) - 30mg

[13187C](https://www.pbs.gov.au/medicine/item/13187c) - 45mg

[8707G](https://www.pbs.gov.au/medicine/item/8707g) - 7.5mg

Leuprorelin & bicalutamide combined

[10962Y](https://www.pbs.gov.au/medicine/item/10962y) - 7.5mg syringe/50mg, 28

[10963B](https://www.pbs.gov.au/medicine/item/10963b) - 22.5mg/50mg, 28

[10969H](https://www.pbs.gov.au/medicine/item/10969h) - 22.5mg/50mg, 84

Lorlatinib

[12096P](https://www.pbs.gov.au/medicine/item/12096p) - 25mg, 90

[12091J](https://www.pbs.gov.au/medicine/item/12091j) - 100mg, 30

Melphalan

[2547C](https://www.pbs.gov.au/medicine/item/2547c) - 2mg, 25

Mercaptopurine

[10214N](https://www.pbs.gov.au/medicine/item/10214n) - 20mg/mL, 100mL oral

[1598D](https://www.pbs.gov.au/medicine/item/1598d) - 50mg, 25

Methotrexate

[11275K](https://www.pbs.gov.au/medicine/item/11275k) - 7.5mg/0.15mL syringe

[11283W](https://www.pbs.gov.au/medicine/item/11283w) - 10mg/0.2mL

[11268C](https://www.pbs.gov.au/medicine/item/11268c) - 15mg/0.3mL

[11288D](https://www.pbs.gov.au/medicine/item/11288d) - 20mg/0.4mL

[11295L](https://www.pbs.gov.au/medicine/item/11295l) - 25mg/0.5mL

[2396D](https://www.pbs.gov.au/medicine/item/2396d) - 5mg/2mLx5 vials

[2395C](https://www.pbs.gov.au/medicine/item/2395c) - 50mg/2mLx5 vials

[4502Y](https://www.pbs.gov.au/medicine/item/4502y), [4512L](https://www.pbs.gov.au/medicine/item/4512l), [7250N](https://www.pbs.gov.au/medicine/item/7250n), [7251P](https://www.pbs.gov.au/medicine/item/7251p) - 1g/10mL, 500mg/20mL, 5g/50mL, 5mg/2mLx5, 50mg/2mLx5 vials for injection

Midostaurin

[11506N](https://www.pbs.gov.au/medicine/item/11506n), [11553C](https://www.pbs.gov.au/medicine/item/11553c) - 25mg, 56

[11505M](https://www.pbs.gov.au/medicine/item/11505m), [11518F](https://www.pbs.gov.au/medicine/item/11518f), [11531X](https://www.pbs.gov.au/medicine/item/11531x), [11552B](https://www.pbs.gov.au/medicine/item/11552b) - 25mg, 112

Nab-paclitaxel

[10150F](https://www.pbs.gov.au/medicine/item/10150f), [10165B](https://www.pbs.gov.au/medicine/item/10165b), [4531L](https://www.pbs.gov.au/medicine/item/4531l), [7270P](https://www.pbs.gov.au/medicine/item/7270p) - 100mg injection

Nilotinib

[12868G](https://www.pbs.gov.au/medicine/item/12868g), [1309X](https://www.pbs.gov.au/medicine/item/1309x) - 150mg, 120

[12858R](https://www.pbs.gov.au/medicine/item/12858r), [12867F](https://www.pbs.gov.au/medicine/item/12867f), [12885E](https://www.pbs.gov.au/medicine/item/12885e), [12887G](https://www.pbs.gov.au/medicine/item/12887g), [9171Q](https://www.pbs.gov.au/medicine/item/9171q) - 200mg, 120

Niraparib

[13089X](https://www.pbs.gov.au/medicine/item/13089x), [**13112D**](https://www.pbs.gov.au/medicine/item/13112d) - 100mg, 56

[**13079J**](https://www.pbs.gov.au/medicine/item/13079j)**,** [13092C](https://www.pbs.gov.au/medicine/item/13092c) - 100mg, 84

Nivolumab

[10745M](https://www.pbs.gov.au/medicine/item/10745m), [10748Q](https://www.pbs.gov.au/medicine/item/10748q), [10764M](https://www.pbs.gov.au/medicine/item/10764m), [10775D](https://www.pbs.gov.au/medicine/item/10775d), [11143L](https://www.pbs.gov.au/medicine/item/11143l), [11150W](https://www.pbs.gov.au/medicine/item/11150w), [11152Y](https://www.pbs.gov.au/medicine/item/11152y), [11153B](https://www.pbs.gov.au/medicine/item/11153b), [11157F](https://www.pbs.gov.au/medicine/item/11157f), [11158G](https://www.pbs.gov.au/medicine/item/11158g), [11159H](https://www.pbs.gov.au/medicine/item/11159h), [11160J](https://www.pbs.gov.au/medicine/item/11160j), [11411N](https://www.pbs.gov.au/medicine/item/11411n), [11425H](https://www.pbs.gov.au/medicine/item/11425h), [11434T](https://www.pbs.gov.au/medicine/item/11434t), [11435W](https://www.pbs.gov.au/medicine/item/11435w), [11532Y](https://www.pbs.gov.au/medicine/item/11532y), [11543M](https://www.pbs.gov.au/medicine/item/11543m), [11626X](https://www.pbs.gov.au/medicine/item/11626x), [11627Y](https://www.pbs.gov.au/medicine/item/11627y), [11636K](https://www.pbs.gov.au/medicine/item/11636k), [11642R](https://www.pbs.gov.au/medicine/item/11642r), [11900H](https://www.pbs.gov.au/medicine/item/11900h), [11906P](https://www.pbs.gov.au/medicine/item/11906p), [12315E](https://www.pbs.gov.au/medicine/item/12315e), [12323N](https://www.pbs.gov.au/medicine/item/12323n), [12574T](https://www.pbs.gov.au/medicine/item/12574t), [12602G](https://www.pbs.gov.au/medicine/item/12602g), [13117J](https://www.pbs.gov.au/medicine/item/13117j), [13121N](https://www.pbs.gov.au/medicine/item/13121n), [**13240W**](https://www.pbs.gov.au/medicine/item/13240w)**,** [**13246E**](https://www.pbs.gov.au/medicine/item/13246e) - 40mg/4mL, 100mg/10mL

Obinutuzumab

[10407R](https://www.pbs.gov.au/medicine/item/10407r), [10418H](https://www.pbs.gov.au/medicine/item/10418h), [11455X](https://www.pbs.gov.au/medicine/item/11455x), [11456Y](https://www.pbs.gov.au/medicine/item/11456y), [11457B](https://www.pbs.gov.au/medicine/item/11457b), [11458C](https://www.pbs.gov.au/medicine/item/11458c), [11460E](https://www.pbs.gov.au/medicine/item/11460e), [11462G](https://www.pbs.gov.au/medicine/item/11462g), [11468N](https://www.pbs.gov.au/medicine/item/11468n), [11473W](https://www.pbs.gov.au/medicine/item/11473w), [12193R](https://www.pbs.gov.au/medicine/item/12193r), [12204H](https://www.pbs.gov.au/medicine/item/12204h) - 1g/40mL

Octreotide LAR (long-acting formulation)

[10543X](https://www.pbs.gov.au/medicine/item/10543x), [10566D](https://www.pbs.gov.au/medicine/item/10566d), [11501H](https://www.pbs.gov.au/medicine/item/11501h) - 10mg MR injection

[10533J](https://www.pbs.gov.au/medicine/item/10533j), [10549F](https://www.pbs.gov.au/medicine/item/10549f), [11537F](https://www.pbs.gov.au/medicine/item/11537f) - 20mg

[10550G](https://www.pbs.gov.au/medicine/item/10550g), [10558Q](https://www.pbs.gov.au/medicine/item/10558q), [11512X](https://www.pbs.gov.au/medicine/item/11512x), [11893Y](https://www.pbs.gov.au/medicine/item/11893y), [11894B](https://www.pbs.gov.au/medicine/item/11894b), [11896D](https://www.pbs.gov.au/medicine/item/11896d) - 30mg

[6227R](https://www.pbs.gov.au/medicine/item/6227r), [9508K](https://www.pbs.gov.au/medicine/item/9508k) - 50mcg/mL x5 ampoules

[6228T](https://www.pbs.gov.au/medicine/item/6228t), [9509L](https://www.pbs.gov.au/medicine/item/9509l) - 100mcg/mL x5

[6229W](https://www.pbs.gov.au/medicine/item/6229w), [9510M](https://www.pbs.gov.au/medicine/item/9510m) - 500mcg/mL x5

Olaparib

[11503K](https://www.pbs.gov.au/medicine/item/11503k), [11522K](https://www.pbs.gov.au/medicine/item/11522k), [12169L](https://www.pbs.gov.au/medicine/item/12169l), [12170M](https://www.pbs.gov.au/medicine/item/12170m), [12921C](https://www.pbs.gov.au/medicine/item/12921c), [12932P](https://www.pbs.gov.au/medicine/item/12932p) - 100mg, 56

[11528R](https://www.pbs.gov.au/medicine/item/11528r), [11539H](https://www.pbs.gov.au/medicine/item/11539h), [12157W](https://www.pbs.gov.au/medicine/item/12157w), [12161C](https://www.pbs.gov.au/medicine/item/12161c), [12913P](https://www.pbs.gov.au/medicine/item/12913p), [12929L](https://www.pbs.gov.au/medicine/item/12929l) - 150mg, 56

Osimertinib

[11620N](https://www.pbs.gov.au/medicine/item/11620n), [12233W](https://www.pbs.gov.au/medicine/item/12233w) - 40mg, 30

[11622Q](https://www.pbs.gov.au/medicine/item/11622q), [12232T](https://www.pbs.gov.au/medicine/item/12232t) - 80mg, 30

Oxaliplatin

[4542C](https://www.pbs.gov.au/medicine/item/4542c), [7253R](https://www.pbs.gov.au/medicine/item/7253r) - 100mg/20mL, 200mg/40mL

Paclitaxel

[4567J](https://www.pbs.gov.au/medicine/item/4567j), [7254T](https://www.pbs.gov.au/medicine/item/7254t) - 300mg/50mL injection

Palbociclib

[12818P](https://www.pbs.gov.au/medicine/item/12818p) - 75mg, 21

[12819Q](https://www.pbs.gov.au/medicine/item/12819q) - 100mg, 21

[12822W](https://www.pbs.gov.au/medicine/item/12822w) - 125mg, 21

Pamidronate

[5667G](https://www.pbs.gov.au/medicine/item/5667g), [6286W](https://www.pbs.gov.au/medicine/item/6286w), [8461H](https://www.pbs.gov.au/medicine/item/8461h) - 15mg/5mL

[5668H](https://www.pbs.gov.au/medicine/item/5668h), [6287X](https://www.pbs.gov.au/medicine/item/6287x), [8462J](https://www.pbs.gov.au/medicine/item/8462j) - 30mg/10mL

[5669J](https://www.pbs.gov.au/medicine/item/5669j), [6288Y](https://www.pbs.gov.au/medicine/item/6288y), [8463K](https://www.pbs.gov.au/medicine/item/8463k) - 60mg/10mL

[5670K](https://www.pbs.gov.au/medicine/item/5670k), [6289B](https://www.pbs.gov.au/medicine/item/6289b) - 90mg/10mL

Panitumumab

[10069Y](https://www.pbs.gov.au/medicine/item/10069y), [10082P](https://www.pbs.gov.au/medicine/item/10082p), [10508C](https://www.pbs.gov.au/medicine/item/10508c), [10513H](https://www.pbs.gov.au/medicine/item/10513h) - 100mg/5mL, 400mg/20mL

Pazopanib

[10054E](https://www.pbs.gov.au/medicine/item/10054e), [2232L](https://www.pbs.gov.au/medicine/item/2232l) - 200mg, 30

[10042M](https://www.pbs.gov.au/medicine/item/10042m), [10047T](https://www.pbs.gov.au/medicine/item/10047t), [11252F](https://www.pbs.gov.au/medicine/item/11252f), [2029T](https://www.pbs.gov.au/medicine/item/2029t) - 200mg, 90

[10052C](https://www.pbs.gov.au/medicine/item/10052c), [2201W](https://www.pbs.gov.au/medicine/item/2201w) - 400mg, 30

[10041L](https://www.pbs.gov.au/medicine/item/10041l), [10043N](https://www.pbs.gov.au/medicine/item/10043n), [11261Q](https://www.pbs.gov.au/medicine/item/11261q), [2030W](https://www.pbs.gov.au/medicine/item/2030w) - 400mg, 60

Pembrolizumab

[10424P](https://www.pbs.gov.au/medicine/item/10424p), [10436G](https://www.pbs.gov.au/medicine/item/10436g), [10475H](https://www.pbs.gov.au/medicine/item/10475h), [10493G](https://www.pbs.gov.au/medicine/item/10493g), [11330H](https://www.pbs.gov.au/medicine/item/11330h), [11352L](https://www.pbs.gov.au/medicine/item/11352l), [11492W](https://www.pbs.gov.au/medicine/item/11492w), [11494Y](https://www.pbs.gov.au/medicine/item/11494y), [11632F](https://www.pbs.gov.au/medicine/item/11632f), [11646Y](https://www.pbs.gov.au/medicine/item/11646y), [12119W](https://www.pbs.gov.au/medicine/item/12119w), [12120X](https://www.pbs.gov.au/medicine/item/12120x), [12121Y](https://www.pbs.gov.au/medicine/item/12121y), [12122B](https://www.pbs.gov.au/medicine/item/12122b), [12123C](https://www.pbs.gov.au/medicine/item/12123c), [12124D](https://www.pbs.gov.au/medicine/item/12124d), [12125E](https://www.pbs.gov.au/medicine/item/12125e),

[12126F](https://www.pbs.gov.au/medicine/item/12126f), [12127G](https://www.pbs.gov.au/medicine/item/12127g), [12128H](https://www.pbs.gov.au/medicine/item/12128h), [12129J](https://www.pbs.gov.au/medicine/item/12129j), [12130K](https://www.pbs.gov.au/medicine/item/12130k), [12605K](https://www.pbs.gov.au/medicine/item/12605k), [12615Y](https://www.pbs.gov.au/medicine/item/12615y), [13114F](https://www.pbs.gov.au/medicine/item/13114f), [13131D](https://www.pbs.gov.au/medicine/item/13131d), [**13254N**](https://www.pbs.gov.au/medicine/item/13254n)**,** [**13267G**](https://www.pbs.gov.au/medicine/item/13267g)**,** [**13286G**](https://www.pbs.gov.au/medicine/item/13286g)**,** [**13287H**](https://www.pbs.gov.au/medicine/item/13287h)**,** [**13608F**](https://www.pbs.gov.au/medicine/item/13608f)**,** [**13626E**](https://www.pbs.gov.au/medicine/item/13626e)**,** [**13635P**](https://www.pbs.gov.au/medicine/item/13635p)**,** [**13645E**](https://www.pbs.gov.au/medicine/item/13645e) - 100mg/4mL

Pemetrexed

[4600D](https://www.pbs.gov.au/medicine/item/4600d), [7255W](https://www.pbs.gov.au/medicine/item/7255w) - 100mg/4mL, 500mg/20mL, 1g/40mL, 1g, 100mg, 500mg injections

Pertuzumab

[10267J](https://www.pbs.gov.au/medicine/item/10267j), [10308M](https://www.pbs.gov.au/medicine/item/10308m), [10333W](https://www.pbs.gov.au/medicine/item/10333w), [10334X](https://www.pbs.gov.au/medicine/item/10334x) - 420mg/14mL

Pomalidomide

[12666P](https://www.pbs.gov.au/medicine/item/12666p), [12668R](https://www.pbs.gov.au/medicine/item/12668r) - 3mg, 14

[10406Q](https://www.pbs.gov.au/medicine/item/10406q), [10417G](https://www.pbs.gov.au/medicine/item/10417g) - 3mg, 21

[12661J](https://www.pbs.gov.au/medicine/item/12661j), [12665N](https://www.pbs.gov.au/medicine/item/12665n) - 4mg, 14

[10386P](https://www.pbs.gov.au/medicine/item/10386p), [10387Q](https://www.pbs.gov.au/medicine/item/10387q) - 4mg, 21

Ponatinib

[10520Q](https://www.pbs.gov.au/medicine/item/10520q), [10523W](https://www.pbs.gov.au/medicine/item/10523w), [11454W](https://www.pbs.gov.au/medicine/item/11454w) - 15mg, 60

[10524X](https://www.pbs.gov.au/medicine/item/10524x), [10530F](https://www.pbs.gov.au/medicine/item/10530f), [11453T](https://www.pbs.gov.au/medicine/item/11453t) - 45mg, 30

Pralatrexate

[11271F](https://www.pbs.gov.au/medicine/item/11271f), [11272G](https://www.pbs.gov.au/medicine/item/11272g), [11278N](https://www.pbs.gov.au/medicine/item/11278n), [11293J](https://www.pbs.gov.au/medicine/item/11293j) - 20mg/mL

Raltitrexed

[4610P](https://www.pbs.gov.au/medicine/item/4610p), [7256X](https://www.pbs.gov.au/medicine/item/7256x) - 2mg injection

Ribociclib

[11385F](https://www.pbs.gov.au/medicine/item/11385f) - 200mg, 21

[11397W](https://www.pbs.gov.au/medicine/item/11397w) - 200mg, 42

[11386G](https://www.pbs.gov.au/medicine/item/11386g) - 200mg, 63

Ripretinib

[12764T](https://www.pbs.gov.au/medicine/item/12764t) - 50mg, 90

Rituximab

[13088W](https://www.pbs.gov.au/medicine/item/13088w), [13090Y](https://www.pbs.gov.au/medicine/item/13090y), [13102N](https://www.pbs.gov.au/medicine/item/13102n), [**13109Y**](https://www.pbs.gov.au/medicine/item/13109y) - 100mg/10mL

[13082M](https://www.pbs.gov.au/medicine/item/13082m), [13090Y](https://www.pbs.gov.au/medicine/item/13090y), [13096G](https://www.pbs.gov.au/medicine/item/13096g), [13102N](https://www.pbs.gov.au/medicine/item/13102n) - 100mg/10mL x2

[13090Y](https://www.pbs.gov.au/medicine/item/13090y), [13095F](https://www.pbs.gov.au/medicine/item/13095f), [13101M](https://www.pbs.gov.au/medicine/item/13101m), [13102N](https://www.pbs.gov.au/medicine/item/13102n) - 500mg/50mL

Ruxolitinib

[10614P](https://www.pbs.gov.au/medicine/item/10614p), [10616R](https://www.pbs.gov.au/medicine/item/10616r), [**13238R**](https://www.pbs.gov.au/medicine/item/13238r)**,** [**13239T**](https://www.pbs.gov.au/medicine/item/13239t)**,** [**13241X**](https://www.pbs.gov.au/medicine/item/13241x)**,** [**13243B**](https://www.pbs.gov.au/medicine/item/13243b)**,** [**13244C**](https://www.pbs.gov.au/medicine/item/13244c) - 5mg, 56

[10913J](https://www.pbs.gov.au/medicine/item/10913j), [10927D](https://www.pbs.gov.au/medicine/item/10927d), [**13231J**](https://www.pbs.gov.au/medicine/item/13231j)**,** [**13232K**](https://www.pbs.gov.au/medicine/item/13232k)**,** [**13235N**](https://www.pbs.gov.au/medicine/item/13235n)**,** [**13236P**](https://www.pbs.gov.au/medicine/item/13236p)**,** [**13245D**](https://www.pbs.gov.au/medicine/item/13245d) - 10mg, 56

[10615Q](https://www.pbs.gov.au/medicine/item/10615q), [10619X](https://www.pbs.gov.au/medicine/item/10619x) - 15mg, 56

[10617T](https://www.pbs.gov.au/medicine/item/10617t), [10618W](https://www.pbs.gov.au/medicine/item/10618w) - 20mg, 56

Sacituzumab govitecan

[12944G](https://www.pbs.gov.au/medicine/item/12944g), [12945H](https://www.pbs.gov.au/medicine/item/12945h), [12965J](https://www.pbs.gov.au/medicine/item/12965j), [12966K](https://www.pbs.gov.au/medicine/item/12966k) - 180mg

Selinexor

[13085Q](https://www.pbs.gov.au/medicine/item/13085q), [13099K](https://www.pbs.gov.au/medicine/item/13099k) - 20mg, 16

[13086R](https://www.pbs.gov.au/medicine/item/13086r), [13103P](https://www.pbs.gov.au/medicine/item/13103p) - 20mg, 20

[13104Q](https://www.pbs.gov.au/medicine/item/13104q), [13105R](https://www.pbs.gov.au/medicine/item/13105r) - 20mg, 32

Sonidegib

[11304Y](https://www.pbs.gov.au/medicine/item/11304y) - 200mg, 30

Sorafenib

[10226F](https://www.pbs.gov.au/medicine/item/10226f), [10242C](https://www.pbs.gov.au/medicine/item/10242c), [9380Q](https://www.pbs.gov.au/medicine/item/9380q) - 200mg, 60

Sunitinib

[10004M](https://www.pbs.gov.au/medicine/item/10004m), [10009T](https://www.pbs.gov.au/medicine/item/10009t), [11266Y](https://www.pbs.gov.au/medicine/item/11266y), [9417P](https://www.pbs.gov.au/medicine/item/9417p), [9420T](https://www.pbs.gov.au/medicine/item/9420t), [9488J](https://www.pbs.gov.au/medicine/item/9488j) - 12.5mg, 28

[11253G](https://www.pbs.gov.au/medicine/item/11253g), [2842N](https://www.pbs.gov.au/medicine/item/2842n), [2959R](https://www.pbs.gov.au/medicine/item/2959r), [9418Q](https://www.pbs.gov.au/medicine/item/9418q), [9421W](https://www.pbs.gov.au/medicine/item/9421w), [9489K](https://www.pbs.gov.au/medicine/item/9489k) - 25mg, 28

[10459L](https://www.pbs.gov.au/medicine/item/10459l), [10464R](https://www.pbs.gov.au/medicine/item/10464r), [10473F](https://www.pbs.gov.au/medicine/item/10473f), [10503T](https://www.pbs.gov.au/medicine/item/10503t), [10504W](https://www.pbs.gov.au/medicine/item/10504w), [11256K](https://www.pbs.gov.au/medicine/item/11256k) - 37.5mg, 28

[10010W](https://www.pbs.gov.au/medicine/item/10010w), [11250D](https://www.pbs.gov.au/medicine/item/11250d), [2837H](https://www.pbs.gov.au/medicine/item/2837h), [9419R](https://www.pbs.gov.au/medicine/item/9419r), [9422X](https://www.pbs.gov.au/medicine/item/9422x), [9490L](https://www.pbs.gov.au/medicine/item/9490l) - 50mg, 28

Tamoxifen

[10911G](https://www.pbs.gov.au/medicine/item/10911g), [1880Y](https://www.pbs.gov.au/medicine/item/1880y) - 20mg, 30

[2110C](https://www.pbs.gov.au/medicine/item/2110c) - 20mg, 60

Temozolomide

[8378Y](https://www.pbs.gov.au/medicine/item/8378y), [8819E](https://www.pbs.gov.au/medicine/item/8819e) - 5mg, 5

[8379B](https://www.pbs.gov.au/medicine/item/8379b), [8820F](https://www.pbs.gov.au/medicine/item/8820f) - 20mg, 5

[8380C](https://www.pbs.gov.au/medicine/item/8380c), [8821G](https://www.pbs.gov.au/medicine/item/8821g) - 100mg, 5

[9361Q](https://www.pbs.gov.au/medicine/item/9361q), [9362R](https://www.pbs.gov.au/medicine/item/9362r) - 140mg, 5

[10062N](https://www.pbs.gov.au/medicine/item/10062n), [2438H](https://www.pbs.gov.au/medicine/item/2438h) - 180mg, 5

[8381D](https://www.pbs.gov.au/medicine/item/8381d) - 250mg, 5

Tepotinib

[13171F](https://www.pbs.gov.au/medicine/item/13171f) - 225mg, 60

Thalidomide

[6469L](https://www.pbs.gov.au/medicine/item/6469l), [9566L](https://www.pbs.gov.au/medicine/item/9566l) - 50mg, 28

[9667T](https://www.pbs.gov.au/medicine/item/9667t), [9684Q](https://www.pbs.gov.au/medicine/item/9684q) - 100mg, 28

Tioguanine

[1233X](https://www.pbs.gov.au/medicine/item/1233x) - 40mg, 25

Topotecan

[4617B](https://www.pbs.gov.au/medicine/item/4617b), [7260D](https://www.pbs.gov.au/medicine/item/7260d) - 4mg/4mL x5, 4mg injections

Trabectedin

[**13340D**](https://www.pbs.gov.au/medicine/item/13340d)**,** [**13344H**](https://www.pbs.gov.au/medicine/item/13344h)**,** [**13346K**](https://www.pbs.gov.au/medicine/item/13346k)**,** [**13348M**](https://www.pbs.gov.au/medicine/item/13348m) - 1mg injection

Trametinib

[10385N](https://www.pbs.gov.au/medicine/item/10385n), [10403M](https://www.pbs.gov.au/medicine/item/10403m), [11821E](https://www.pbs.gov.au/medicine/item/11821e) - 500mcg, 30

[10382K](https://www.pbs.gov.au/medicine/item/10382k), [10405P](https://www.pbs.gov.au/medicine/item/10405p), [11819C](https://www.pbs.gov.au/medicine/item/11819c) - 2mg, 30

Trastuzumab

[10682F](https://www.pbs.gov.au/medicine/item/10682f), [10743K](https://www.pbs.gov.au/medicine/item/10743k), [10798H](https://www.pbs.gov.au/medicine/item/10798h), [10803N](https://www.pbs.gov.au/medicine/item/10803n), [10811B](https://www.pbs.gov.au/medicine/item/10811b), [10817H](https://www.pbs.gov.au/medicine/item/10817h) - 600mg/5mL

[10383L](https://www.pbs.gov.au/medicine/item/10383l), [10391X](https://www.pbs.gov.au/medicine/item/10391x), [10401K](https://www.pbs.gov.au/medicine/item/10401k), [10402L](https://www.pbs.gov.au/medicine/item/10402l), [10581X](https://www.pbs.gov.au/medicine/item/10581x), [10588G](https://www.pbs.gov.au/medicine/item/10588g), [10589H](https://www.pbs.gov.au/medicine/item/10589h), [10597R](https://www.pbs.gov.au/medicine/item/10597r), [4632T](https://www.pbs.gov.au/medicine/item/4632t), [4639E](https://www.pbs.gov.au/medicine/item/4639e), [4650R](https://www.pbs.gov.au/medicine/item/4650r), [4703M](https://www.pbs.gov.au/medicine/item/4703m), [7264H](https://www.pbs.gov.au/medicine/item/7264h), [7265J](https://www.pbs.gov.au/medicine/item/7265j), [7266K](https://www.pbs.gov.au/medicine/item/7266k), [7267L](https://www.pbs.gov.au/medicine/item/7267l) - 420mg, 440mg, 150mg, 60mg

Trastuzumab deruxtecan

[**13713R**](https://www.pbs.gov.au/medicine/item/13713r)**,** [**13718B**](https://www.pbs.gov.au/medicine/item/13718b) - 100mg injection

Trastuzumab emtansine

[10281D](https://www.pbs.gov.au/medicine/item/10281d), [10282E](https://www.pbs.gov.au/medicine/item/10282e), [11951B](https://www.pbs.gov.au/medicine/item/11951b), [11956G](https://www.pbs.gov.au/medicine/item/11956g) - 100mg, 160mg

Trifluridine/tipiracil

[11507P](https://www.pbs.gov.au/medicine/item/11507p), [12056M](https://www.pbs.gov.au/medicine/item/12056m) - 15mg/6.14mg, 20

[11524M](https://www.pbs.gov.au/medicine/item/11524m), [12033H](https://www.pbs.gov.au/medicine/item/12033h) - 20mg/8.19mg, 20

Triptorelin

[9379P](https://www.pbs.gov.au/medicine/item/9379p) - 11.25mg

[5297T](https://www.pbs.gov.au/medicine/item/5297t) - 22.5mg

[9378N](https://www.pbs.gov.au/medicine/item/9378n) - 3.75mg

Vemurafenib

[11076Y](https://www.pbs.gov.au/medicine/item/11076y), [11081F](https://www.pbs.gov.au/medicine/item/11081f) - 240mg, 56

Venetoclax

[12999E](https://www.pbs.gov.au/medicine/item/12999e) - 10mg, 2

[11630D](https://www.pbs.gov.au/medicine/item/11630d), [12188L](https://www.pbs.gov.au/medicine/item/12188l) - 10mg, 14; 50mg, 7; 100mg, 7; 100mg, 14

[11648C](https://www.pbs.gov.au/medicine/item/11648c), [12773G](https://www.pbs.gov.au/medicine/item/12773g) - 50mg, 7

[11639N](https://www.pbs.gov.au/medicine/item/11639n), [12199C](https://www.pbs.gov.au/medicine/item/12199c), [12205J](https://www.pbs.gov.au/medicine/item/12205j), [12803W](https://www.pbs.gov.au/medicine/item/12803w) - 100mg, 120

Vinblastine

[4618C](https://www.pbs.gov.au/medicine/item/4618c), [7261E](https://www.pbs.gov.au/medicine/item/7261e) - 10mg/10mL x5

Vincristine

[4619D](https://www.pbs.gov.au/medicine/item/4619d), [7262F](https://www.pbs.gov.au/medicine/item/7262f) - 1mg/mL x5

Vinorelbine

[9009E](https://www.pbs.gov.au/medicine/item/9009e) - 20mg, 1

[9010F](https://www.pbs.gov.au/medicine/item/9010f) - 30mg, 1

[4620E](https://www.pbs.gov.au/medicine/item/4620e), [7263G](https://www.pbs.gov.au/medicine/item/7263g) - 10mg/mL, 50mg/5mL

Vismodegib

[11070P](https://www.pbs.gov.au/medicine/item/11070p) - 150mg, 28

Vorinostat

[11138F](https://www.pbs.gov.au/medicine/item/11138f), [11141J](https://www.pbs.gov.au/medicine/item/11141j) - 100mg, 120

Zanubrutinib

[12891L](https://www.pbs.gov.au/medicine/item/12891l), [13041J](https://www.pbs.gov.au/medicine/item/13041j), [**13616P**](https://www.pbs.gov.au/medicine/item/13616p)**,** [**13628G**](https://www.pbs.gov.au/medicine/item/13628g) - 80mg, 120

Zoledronic acid

[6371H](https://www.pbs.gov.au/medicine/item/6371h), [9653C](https://www.pbs.gov.au/medicine/item/9653c) - 4mg/5mL

[9288W](https://www.pbs.gov.au/medicine/item/9288w), [9350D](https://www.pbs.gov.au/medicine/item/9350d) - 5mg/100mL

*Ceritinib

[11056X](https://www.pbs.gov.au/medicine/item/11056x) - 150mg, 3x50

*Crizotinib

[10323H](https://www.pbs.gov.au/medicine/item/10323h), [11589Y](https://www.pbs.gov.au/medicine/item/11589y) - 200mg, 60

[10322G](https://www.pbs.gov.au/medicine/item/10322g), [11594F](https://www.pbs.gov.au/medicine/item/11594f) - 250mg, 60

*Erlotinib

[10022L](https://www.pbs.gov.au/medicine/item/10022l), [10028T](https://www.pbs.gov.au/medicine/item/10028t), [11263T](https://www.pbs.gov.au/medicine/item/11263t) - 25mg, 30

[10019H](https://www.pbs.gov.au/medicine/item/10019h), [10020J](https://www.pbs.gov.au/medicine/item/10020j), [11260P](https://www.pbs.gov.au/medicine/item/11260p) - 100mg, 30

[10014C](https://www.pbs.gov.au/medicine/item/10014c), [10025P](https://www.pbs.gov.au/medicine/item/10025p), [11259N](https://www.pbs.gov.au/medicine/item/11259n) - 150mg, 30

*Flutamide

[1417N](https://www.pbs.gov.au/medicine/item/1417n) - 250mg, 100

*Gefitinib

[11264W](https://www.pbs.gov.au/medicine/item/11264w), [8769M](https://www.pbs.gov.au/medicine/item/8769m) - 250mg, 30

**Aflibercept

[10505X](https://www.pbs.gov.au/medicine/item/10505x), [11991D](https://www.pbs.gov.au/medicine/item/11991d), [12131L](https://www.pbs.gov.au/medicine/item/12131l), [13138L](https://www.pbs.gov.au/medicine/item/13138l), [13146X](https://www.pbs.gov.au/medicine/item/13146x), [13151E](https://www.pbs.gov.au/medicine/item/13151e), [13164W](https://www.pbs.gov.au/medicine/item/13164w), [2168D](https://www.pbs.gov.au/medicine/item/2168d) - 4mg/0.1mL vial

[12132M](https://www.pbs.gov.au/medicine/item/12132m), [12141B](https://www.pbs.gov.au/medicine/item/12141b), [12152N](https://www.pbs.gov.au/medicine/item/12152n), [12153P](https://www.pbs.gov.au/medicine/item/12153p), [13139M](https://www.pbs.gov.au/medicine/item/13139m), [13141P](https://www.pbs.gov.au/medicine/item/13141p), [13150D](https://www.pbs.gov.au/medicine/item/13150d), [13167B](https://www.pbs.gov.au/medicine/item/13167b) - 3.6mg/0.09mL syringe

**Mitoxantrone

[4514N](https://www.pbs.gov.au/medicine/item/4514n), [7252Q](https://www.pbs.gov.au/medicine/item/7252q) - 20mg/10mL, 25mg/12.5mL injections

**Ofatumumab

[12641H](https://www.pbs.gov.au/medicine/item/12641h), [12642J](https://www.pbs.gov.au/medicine/item/12642j) - 20mg/0.4mL pen

Non-PBS drugs listed in eviQ (limitations):

*SUPERSEDED

**part of a DISCONTINUED protocol only

Dacarbazine

- not TGA approved or PBS listed

Dactinomycin

- TGA registered but not PBS listed

Daunorubicin

- TGA registered but not PBS listed

Ixazomib

- TGA registered but not PBS listed

Lomustine

- TGA registered but not PBS listed for this indication

Mitomycin

- TGA registered but not PBS listed

Mitotane

- neither TGA registered or PBS listed for this indication

Pegaspargase

- TGA registered but not PBS listed for this indication

Polatuzumab vedotin

- TGA registered but not PBS listed

Procarbazine

- TGA registered but not PBS listed

Ramucirumab

- TGA registered but not PBS listed

Regorafenib

- TGA registered but not PBS listed

Relatlimab

- TGA registered but not PBS listed for this indication

Romidepsin

- TGA registered but not PBS listed

Sotorasib

- provisionally TGA approved but not PBS listed

Temsirolimus

- TGA registered but not PBS listed

Thiotepa

- TGA registered but not PBS listed

Trastuzumab deruxtecan

- TGA registered but not PBS listed

Tretinoin (ATRA)

- TGA registered but not PBS listed for this indication

*Asparaginase (colaspase)

- TGA registered but not PBS listed for this indication

*Neratinib

- TGA registered but not PBS listed for this indication

*Teniposide

- removed from the [ARTG](https://www.tga.gov.au/ws-sc-index?search_api_views_fulltext=teniposide&field_sc_decision_status=All&sort_by=field_date&items_per_page=10) in June 2017 due to worldwide production cessation. Access may still be available via the [Special Access Scheme.](https://www.tga.gov.au/form/special-access-scheme)

**Interferon alfa-2a

- not found on PBS

**Interferon alfa-2b

- not found on PBS; discontinuation of product supply

**Nilutamide

- no longer TGA registered, or PBS listed

**Vandetanib

- not found on PBS; causes tdP

**Vinflunine

- no longer available in Australia
